# HCM-Associated MuRF1 Variants Compromise Ubiquitylation and Are Predicted to Alter Protein Structure

**DOI:** 10.3390/ijms26083921

**Published:** 2025-04-21

**Authors:** Jitpisute Chunthorng-Orn, Maya Noureddine, Peter W. J. Dawson, Samuel O. Lord, Jimi Ng, Luke Boyton, Katja Gehmlich, Fiyaz Mohammed, Yu-Chiang Lai

**Affiliations:** 1 School of Sport, Exercise and Rehabilitation Sciences, University of Birmingham, Birmingham B15 2TT, UK or jitpisut@tu.ac.th (J.C.-O.); pwjdawson@gmail.com (P.W.J.D.); lhb949@student.bham.ac.uk (L.B.); 2 Department of Applied Thai Traditional Medicine, Faculty of Medicine, University of Thammasat, Pathumthani 12120, Thailand; 3 Department of Cardiovascular Sciences, School of Medical Sciences, College of Medicine and Health, University of Birmingham, Birmingham B15 2TT, UK; mmn207@student.bham.ac.uk (M.N.); or katja.gehmlich@cardiov.ox.ac.uk (K.G.); 4 Cancer Research UK Cambridge Institute, University of Cambridge, Robinson Way, Cambridge CB2 0RE, UK; sam.lord@cruk.cam.ac.uk; 5 Centre for Systems Health and Integrated Metabolic Research, Department of Biosciences, Nottingham Trent University, Nottingham NG11 8NS, UK; jiming1011@gmail.com; 6 Division of Cardiovascular Medicine, Radcliffe Department of Medicine and British Heart Foundation Centre of Research Excellence Oxford, University of Oxford, Oxford OX3 9DU, UK; 7 Department of Immunology and Immunotherapy, School of Infection, Inflammation and Immunology, College of Medicine and Health, University of Birmingham, Birmingham B15 2TT, UK; 8 MRC Versus Arthritis Centre for Musculoskeletal Ageing Research, University of Birmingham, Birmingham B15 2TT, UK

**Keywords:** MuRF1, *TRIM63*, pathogenic variants, hypertrophic cardiomyopathy, ubiquitylation, structural modelling, MuRF1 domains

## Abstract

MuRF1 [muscle RING (Really Interesting New Gene)-finger protein-1] is an ubiquitin-protein ligase (E3), which encode by *TRIM63* (*tripartite motif containing 63*) gene, playing a crucial role in regulating cardiac muscle size and function through ubiquitylation. Among hypertrophic cardiomyopathy (HCM) patients, 24 *TRIM63* variants have been identified, with 1 additional variant linked to restrictive cardiomyopathy. However, only three variants have been previously investigated for their functional effects. The structural impacts of the 25 variants remain unexplored. This study investigated the effects of 25 MuRF1 variants on ubiquitylation activity using in vitro ubiquitylation assays and structural predictions using computational approaches. The variants were generated using site-directed PCR (Polymerase Chain Reaction) mutagenesis and subsequently purified with amylose affinity chromatography. In vitro ubiquitylation assays demonstrated that all 25 variants compromised the ability of MuRF1 to monoubiquitylate a titin fragment (A168-A170), while 17 variants significantly impaired or completely abolished auto-monoubiquitylation. Structural modelling predicted that 10 MuRF1 variants disrupted zinc binding or key stabilising interactions, compromising structural integrity. In contrast, three variants were predicted to enhance the structural stability of MuRF1, while six others were predicted to have no discernible impact on the structure. This study underscores the importance of functional assays and structural predictions in evaluating MuRF1 variant pathogenicity and provides novel insights into mechanisms by which these variants contribute to HCM and related cardiomyopathies.

## 1. Introduction

Hypertrophic cardiomyopathy (HCM) is recognised as the most common form of inherited cardiomyopathy, with an estimated prevalence of 1:200–1:500 in the general population [1]. The condition is primarily linked to genetic variants in sarcomeric genes; however, emerging evidence has highlighted the involvement of non-sarcomeric genes, including muscle-specific RING (Really Interesting New Gene) finger protein 1 (MuRF1, encoded by the gene *TRIM63; tripartite motif containing 63*), which has demonstrated moderate to strong associations in patients with HCM [2]. Notably, MuRF1 exhibits anti-hypertrophic effects in both in vitro and in vivo settings through the ubiquitylation of sarcomeric proteins [3,4]. In addition, MuRF1 has been identified as a ligand for titin, a substantial protein that spans half of the sarcomere and plays a critical role in regulating the essential functions of striated muscle [5]. This interaction facilitates the ubiquitylation of titin, a process that is vital for maintaining the structural integrity and functionality of titin through its degradation [6].

Ubiquitylation is a post-translation modification process wherein the small protein ubiquitin is covalently attached to a target protein. This modification is mediated by a cascade of three enzymes, namely the ubiquitin-activating enzyme (E1), the ubiquitin-conjugating enzyme (E2), and the ubiquitin-protein ligase (E3, to which MuRF1 belongs) [7]. The ubiquitylation process can be delineated into three main steps. Initially, E1 activates free ubiquitin through adenylation and the formation of a thioester bond. Subsequently, the activated ubiquitin is transferred to E2 before binding to the substrate-conjugated E3. In the final step, RING-type E3 transfers the activated ubiquitin from E2 to the substrate. RING-type E3 represents the majority of E3 ligases, and MuRF1 belongs to this group. In contrast, HECT (homologous to E6AP C-terminus)- and RBR (RING-between-RING)-type E3 ligases first link the activated ubiquitin to the E3 enzyme before transferring it to the substrate [7]. Ubiquitylation encompasses various cellular functions by regulating both proteolytic and non-proteolytic mechanisms, which are contingent on ubiquitylation patterns, including monoubiquitylation, multi-monoubiquitylation, and polyubiquitylation [8].

MuRF1 plays a pivotal role in regulating muscle mass and cardiac muscle function through the ubiquitin–proteasome system (UPS) [9]. Notably, the imbalance of UPS, which is activated downstream of ubiquitylation, has been observed in HCM caused by sarcomeric gene variants in various mouse models and patient studies [10]. These genetic variants often result in proteasomal overload, where the UPS becomes overly engaged in clearing misfolded mutant proteins to perform its normal function of maintaining homeostasis in cardiomyocytes. Additionally, it has been suggested that the lack of ubiquitylation and the turnover of calcineurin A (CnA) activates cardiac hypertrophy through the NFAT (nuclear factor of activated T cells)-CnA signalling pathway, which is inhibited by MuRF1 [3]. MuRF1 exerts anti-hypertrophic effects in both in vitro and in vivo settings by ubiquitylating sarcomeric proteins for degradation [3,10]. For example, the interaction of MuRF1 and titin facilitates the ubiquitylation of titin for degradation, preserving the structural integrity and functionality of titin [6]. MuRF1 also binds to other contractile proteins, such as troponin I, troponin C, and ventricular myosin light chain-2, for degradation via the UPS [11].

In transgenic mice, cardiac-specific MuRF1 overexpression decreased the cross-sectional area of cardiomyocytes following a transaortic construction model [12], while MuRF1 knockout mice showed an aggravated hypertrophic response to biomechanical stress [13]. These findings support the notion that impairments in protein degradation, whether due to an overload of the UPS caused by sarcomeric gene variants or a lack of ubiquitylation associated with MuRF1 variants, lead to the activation of hypertrophy and result in HCM.

Within the UPS framework, MuRF1 facilitates the transfer of activated ubiquitin from ubiquitin-conjugating enzymes (E2s or UBE2s) to specific substrate proteins as an E3 ligase. In vitro ubiquitylation studies have demonstrated that MuRF1 can interact with UBE2W (ubiquitin-conjugating enzyme; E2W) to monoubiquitylate itself (auto-monoubiquitylation; Auto-mUb) or monoubiquitylate His-Titin (A168-A170) (His-Titin-mUb) [14]. The titin A168-A170 segment comprises two immunoglobulin-like (Ig) domains (A168 and A169) and a fibronectin type III (FnIII) domain (A170) (Ig^A168^, Ig^A169^, and Fn III^A170^) located within the A-band of the sarcomere, all of which can interact with MuRF1 [5]. Likewise, the titin-binding motif includes multiple domains of MuRF1, reflecting its structural and functional versatility [6,15].

**Figure 1:**
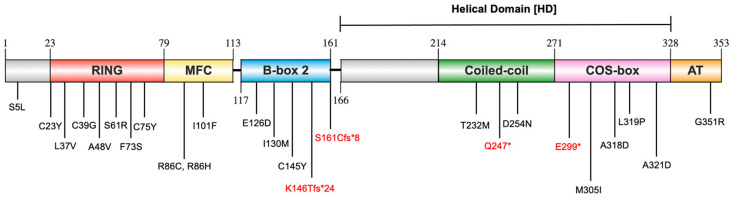
Schematic representation of the MuRF1 [muscle RING (Really Interesting New Gene)-finger protein-1] domain composition and the distribution of HCM (hypertrophic cardiomyopathy)-associated variants. MuRF1 comprises six distinct domains: Really Interesting New Gene (RING) (C23-R79; red), MuRF family-specific motif (MFC) (H80-P113; yellow), B-box type 2 (B-box2) (G117-S161; blue), Coiled-coil (CC) (D214-G271; green), C-terminal subgroup One Signature-box (COS-box) (G272-E328; pink), and Acidic tail (AT) (E329-Q353; orange). Grey regions denote intrinsically disordered domains. The truncated variants are highlighted in red. This figure was generated using IBS 2.0 (https://ibs.renlab.org/#/server, accessed on 1 June–31 July 2024).

This functional versatility is attributed to MuRF1’s six distinct structural domains: Really Interesting New Gene (RING), MuRF family-specific motif (MFC), B-box type 2 (B-box2), Coiled-coil (CC), C-terminal subgroup One Signature-box (COS-box), and Acidic tail (AT) (Figure 1) [16,17,18]. These domains mediate interactions with various proteins, thereby influencing many biological processes. For instance, the RING domain binds to various UBE2s, playing a key role in the ubiquitylation pathway by transferring activated ubiquitin from UBE2 to the targeted substrate [18]. Additionally, the B-box2 and COS-box domains are essential for the correct self-assembly of the CC structure, which is critical for maintaining the structural integrity and functionality of MuRF1 [17]. The CC domain is characterised by the presence of heptad repeats, which are vital for facilitating protein–protein interactions and enabling the formation of dimers or more complex oligomeric structures. Furthermore, studies on skeletal muscle in vivo have indicated that the COS-box is crucial for the recruitment of MuRF1 to the sarcomere [17].

In the last decade, *TRIM63* has been implicated in HCM with 24 distinct variants identified in affected patients (Tables S1 and S2) [9,19,20,21,22,23,24]. Notably, one study reported a *TRIM63* missense variant (C145Y) in patients with restrictive cardiomyopathy (RCM) [23], a condition that shares pathophysiological features, particularly diastolic dysfunction, with HCM. Thus, the C145Y variant was included in this study. Among these 25 variants, only 3 (A48V, I130M, and Q247*) have been previously investigated for their E3 ligase activity [19]. Using adult cardiac myocytes infected with an adenovirus, these three variants compromised E3 ubiquitin ligase activity, resulting in mislocalisation and impaired auto-ubiquitylation and ubiquitylation of MuRF1’s substrates, including cardiac alpha-myosin heavy chain, cardiac myosin-binding protein C, and calcineurin [19]. In transgenic mouse hearts, the expression of these three variants resulted in cardiac hypertrophy, increased mTOR (mammalian target of rapamycin)/S6K1 (Ribosomal protein S6 kinase 1) and calcineurin-RCAN1.4 (regulator of calcineurin 1 isoform 4) signalling, and activated mRNA levels of hypertrophic markers, including ANP (atrial natriuretic peptide), BNP (B-type natriuretic peptide), and β-myosin heavy chain [19].

To date, no additional studies have systematically examined the impact of the remaining 22 variants on ubiquitin activity, nor assessed their effects on MuRF1 structure. Therefore, the present study aims to address these gaps by performing a comprehensive analysis of the functional impact of all 25 identified variants in MuRF1, focusing on in vitro ubiquitin activity (auto-monoubiquitylation) and substrate titin monoubiquitylation. Moreover, this research further aims to predict the structural integrity implications of these variants using computational approaches. This will be the first step towards future research on MuRF1 variants and enhance the understanding of how impaired ubiquitylation and compromised protein structures may contribute to cardiac dysfunction, ultimately leading to cardiomyopathy.

## 2. Results

### 2.1. Distribution of Identified MuRF1 Variants Across MuRF1 Structural Domains

The variants were identified by a search in PubMed using the keywords “TRIM63 HCM” and “MuRF1 HCM” from the inception of the database up to March 2022. The titles and abstracts of the studies identified in the search were first screened, followed by a review of the full texts. In total, seven relevant studies were included in the summary (Tables S1 and S2), which identified 24 *TRIM63* variants in HCM patients and 1 *TRIM63* variant (C145Y) reported in RCM patients. Consequently, the 25 disease-associated *TRIM63* variants were selected based on a review of published papers.

The 25 identified MuRF1 variants are distributed across multiple structural domains of MuRF1, with the majority localised to the RING and MFC domains (Figure 1). Among these variants, 21 are classified as missense mutations, while 2 are categorised as nonsense mutations (Q247* and E299*), and 2 are frameshift mutations (K146Tfs*24 and S161Cfs*8), truncating the protein. Given that only a limited number of these variants have been previously investigated, in vitro ubiquitylation assays were performed to further evaluate their functional impact. Additionally, the structural consequences of MuRF1 variants were predicted using computational prediction tools. Generating accurate structure predictions for truncating variants (K146Tfs*24, S161Cfs*8, Q247*, and E299*) poses challenges for existing molecular modelling tools. Furthermore, the N-terminal (S5L) and AT (G351R) variants reside in regions recognised as intrinsically disordered, rendering their effects on protein structure difficult to model reliably. Consequently, these six variants (S5L, K146Tfs*24, S161Cfs*8, Q247*, E299*, and G351R) were excluded from structural prediction analysis.

### 2.2. The Majority of MuRF1 Variants Impair MuRF1 Auto-Monoubiquitylation

**Figure 2:**
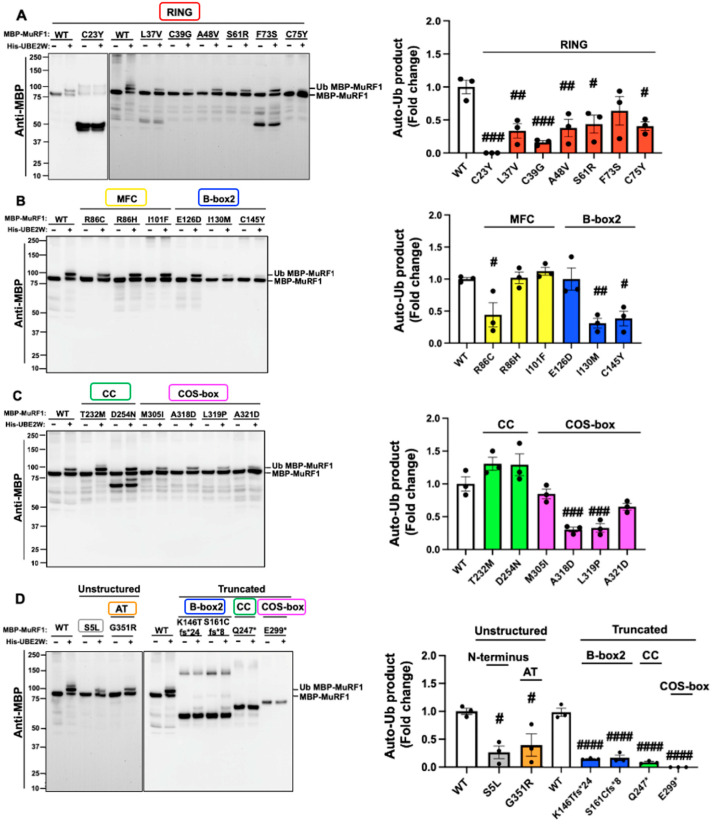
In vitro auto-monoubiquitylation analysis of 25 MuRF1 variants highlights functional impairments. Each reaction included MBP-MuRF1-WT (Maltose-Binding Protein-MuRF1 wild type) or the variant His-UBE1 (Histidine-UBE1) and free ubiquitin and was incubated for one hour with and without His-UBE2W. MBP-MuRF1-WT with His-UBE2W served as a positive control, while reactions without His-UBE2W acted as negative controls for each sample. The experiments with WT MBP-MuRF1, both with and without His-UBE2W, were repeated for each membrane. Ubiquitylation levels were evaluated through immunoblotting using anti-MBP and anti-FK2 (anti-ubiquitylated protein) antibodies. Coomassie blue staining was used to detect His-UBE1, His-UBE2W, and free ubiquitin in the reactions. (**A**) Anti-MBP immunoblot results for MuRF1 variants that map on the RING domain. (**B**) Anti-MBP immunoblot results for MuRF1 variants that map on the MFC and B-box2 domains. (**C**) Anti-MBP immunoblot results for MuRF1 variants that map on the Coiled-coil and COS-box domains. (**D**) Anti-MBP immunoblot results of unstructured and truncated MuRF1 variants. The results of anti-FK2 and Coomassie blue staining are shown in the Supplementary Materials. Densitometry analysis of the Western blot results (anti-MBP) quantifying the ubiquitylation product, as indicated by the upper shifted band. MuRF1-RING, MFC, B-box2, CC, COS-box, N-terminus, and AT variants are shown in red, yellow, blue, green, pink, grey, and orange, respectively. Data were presented as the mean ± SEM (biological replicates = 3), with fold changes calculated relative to the WT. Statistical significance is indicated as follows: ^#^ *p <* 0.05, ^##^ *p* < 0.01, ^###^ *p* < 0.001, and ^####^ *p* < 0.0001. Results were compared to the WT via one-way ANOVA followed by Dunnett’s test.

The interaction between MuRF1-WT (wild type of MuRF1) and UBE2W is a well-documented phenomenon that facilitates MuRF1 monoubiquitylation, referred to as auto-monoubiquitylation (Auto-mUb) [14]. To investigate the effect of MuRF1 variants on Auto-mUb, in vitro ubiquitylation assays were carried out using His-UBE2W (Histidine-UBE2W) and MBP-MuRF1 (Maltose-Binding Protein-MuRF1). These assays were designed to compare the effects of different MBP-MuRF1 variants against MBP-MuRF1-WT, which served as a positive control, while reactions devoid of His-UBE2W functioned as negative controls. The presence of His-UBE1 (Histidine-UBE1), His-UBE2W, and free ubiquitin in each reaction was confirmed through Coomassie blue gel staining (Figure S1). As expected, the results demonstrated that MuRF1-WT with His-UBE2W exhibited clear signs of monoubiquitylation, while no such modification was observed in the absence of His-UBE2W (Figure 2).

A total of 15 MuRF1-variants demonstrated diminished Auto-mUb activity, including S5L, L37V, C39G, A48V, S61R, C75Y, R86C, I130M, C145Y, K146Tfs*24, S161Cfs*8, Q247*, A318D, L319P, and G351R. Two MuRF1 variants, C23Y and E299*, completely abrogated Auto-mUb activity. In contrast, the remaining eight MuRF1 variants (F73S, R86H, I101F, E126D, T232M, D254N, M305I, and A321D) displayed Auto-mUb activities comparable to those of MuRF1-WT (Figure 2). Further analysis revealed that the C23Y variant likely represents a truncated form of MuRF1, as evidenced by a stronger band at ~50 kDa, which is significantly lower than the expected full-length protein size of 83 kDa. Additionally, the variants L37V (RING), F73S (RING), and D254N (CC) displayed partial degradation, appearing at molecular weights of ~50 kDa for L37V and F73S and below 75 kDa for D254N. Intriguingly, the degraded form of D254N retained Auto-mUb activity. Collectively, 17 MuRF1 variants negatively impacted the Auto-mUb process, while the remaining 8 variants exhibited functional characteristics akin to MuRF1-WT (Figure 2).

### 2.3. MuRF1 Variants Preferentially Hinder Monoubiquitylation of the Titin Fragment (A168-A170) Compared to Auto-Monoubiquitylation

**Figure 3:**
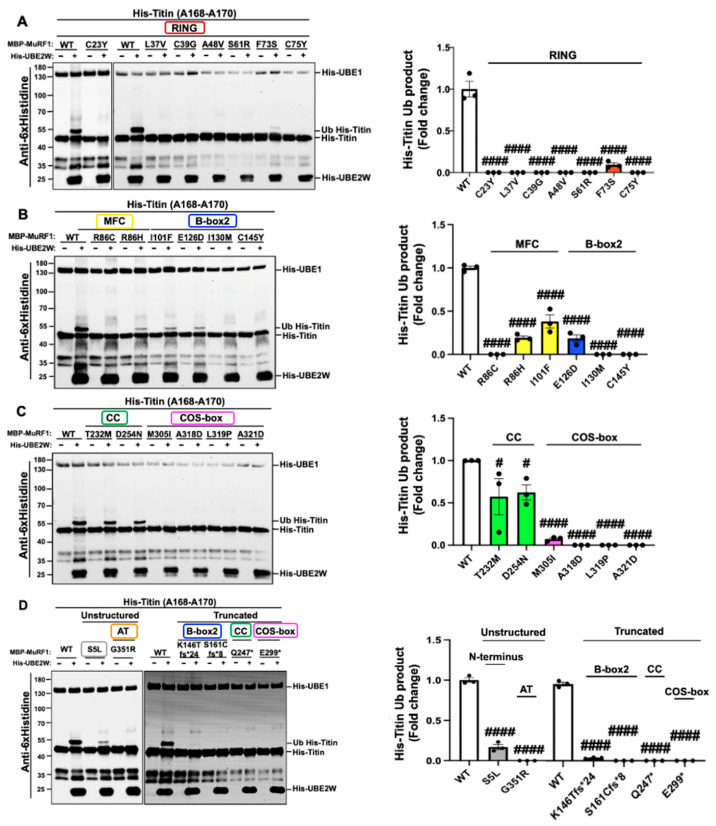
In vitro His-Titin (A168-A170) monoubiquitylation analysis demonstrates that all MuRF1 variants impair MuRF1 function. The assay included wild-type MBP-MuRF1 or the variants His-Titin (A168-A170) and His-UBE1 and free ubiquitin, which were incubated for one hour in the presence and absence of His-UBE2W. wild-type MBP-MuRF1 with His-UBE2W served as a positive control, while the reactions devoid of His-UBE2W acted as negative controls for each sample. The experiments with WT MBP-MuRF1, both with and without His-UBE2W, were repeated for each membrane. Ubiquitylation levels were assessed through immunoblotting using anti-His and anti-FK2 (anti-ubiquitylated protein). Coomassie blue staining was used to detect His-UBE1, His-UBE2W, and free ubiquitin within the reaction. (**A**) Anti-6x Histidine immunoblot results of MuRF1 missense variants that map on the RING domain. (**B**) Anti-6x Histidine immunoblot results of MuRF1 missense variants that map on the MFC and B-box2 domains. (**C**) Anti-6x Histidine immunoblot results of MuRF1 missense variants that map on the Coiled-coil and COS-box domains. (**D**) Anti-6x Histidine immunoblot results of unstructured and truncated MuRF1 variants. The results of anti-FK2 and Coomassie blue staining were shown in the Supplementary Materials. Densitometry analysis of Western blot results (anti-6xHistidine) quantifying the ubiquitylation product, as indicated by the upper shifted band. MuRF1-RING, MFC, B-box2, CC, COS-box, N-terminus, and AT variants are shown in red, yellow, blue, green, pink, grey, and orange, respectively. Data are presented as the mean ± SEM (biological replicates = 3), with fold changes calculated relative to the WT. Statistical significance is indicated as follows ^#^ *p* < 0.05, ^####^ *p* < 0.0001. The results are compared to the WT via one-way ANOVA followed by Dunnett’s test.

In the previous section, we showed that MuRF1 variants exert diverse effects on the MuRF1 Auto-mUb process, which can manifest as a reduction, complete loss, or no change in activity. To further investigate whether the impact of MuRF1 variants on ubiquitylation of an external substrate is consistent, ubiquitylation assays were conducted with the His-Titin (Histidine-Titin) fragment A168-A170 as a substrate for MuRF1. MuRF1 can monoubiquitylate the His-Titin fragment (His-Titin-mUb) in the presence of UBE2W through an in vitro ubiquitylation assay. Coomassie blue gel staining was performed to visualise the presence of His-UBE1, His-UBE2W, and free ubiquitin in each reaction (Figure S2). The results from these assays revealed two main outcomes: a significant reduction or a complete loss of His-Titin-mUb activity. Nine variants, S5L, F73S, R86H, I101F, E126D, K146Tfs*24, T232M, D254N, and M305I, exhibited a marked decrease in His-Titin-mUb activity. Interestingly, seven of these variants (F73S, R86H, I101F, E126D, T232M, D254N, and M305I) did not significantly affect Auto-mUb, indicating a nuanced relationship between these variants and functional outcomes (Figure 3). Notably, 16 variants demonstrated a complete lack of activity on His-Titin-mUb including C23Y, L37V, C39G, A48V, S61R, C75Y, R86C, I130M, C145Y, A161Cfs*8, Q247*, E299*, A318D, L319P, A321D, and G351R (Figure 3). Most of these variants were also found to reduce MuRF1 activity in Auto-mUb, with only two variants (C23Y and E299*) showing consistent effects by exhibiting no activity in either assay (Figure 3). Overall, these findings indicate that His-Titin-mUb was impaired by all 25 MuRF1 variants, suggesting that these variants negatively influence His-Titin-mUb more than Auto-mUb. This observation led us to speculate that MuRF1 variants may compromise structural integrity, disrupting MuRF1 conformations during Auto-mUb and His-Titin-mUb processes, thereby impairing MuRF1 function. Consequently, the impact of MuRF1 variants on protein structure was modelled in the subsequent sections.

### 2.4. Assessment of the MuRF1-RING Domain: Variants Impair Zinc Binding and Compromise Core Interactions Critical for MuRF1’s Structural Integrity

Given the absence of an experimentally derived structure of the MuRF1-RING-MFC domain, we initially generated a molecular model of the MuRF1-RING-MFC domain using AlphaFold2 [25] to investigate the effects of MuRF1 variants on protein structure. Upon superimposing this model onto the crystal structure of a closely related TRIM family member, the TRIM21-RING domain (PDB ID: 8A58) [26], it aligned well [Root Mean Square Deviation (RMSD) of 0.729 Å] (Figure S3A). This finding suggested that the MuRF1-RING-MFC model could reliably be employed for comparative analyses with MuRF1 structures encompassing disease-associated variants. Given that the RING domain is known to bind to UBE2W [26], our initial focus was on assessing the impact of MuRF1-RING variants on UBE2W binding. To this end, we generated a model of the MuRF1-RING-MFC/UBE2W complex using the HADDOCK (High Ambiguity Driven protein-protein Docking) software version 2.4 [27] (Table S4). Notably, none of the variants were found to reside within the predicted UBE2W-binding interface (Figure S3B), suggesting that direct interference with UBE2W binding is an unlikely mechanism underlying the functional impairments associated with these variants.

**Figure 4:**
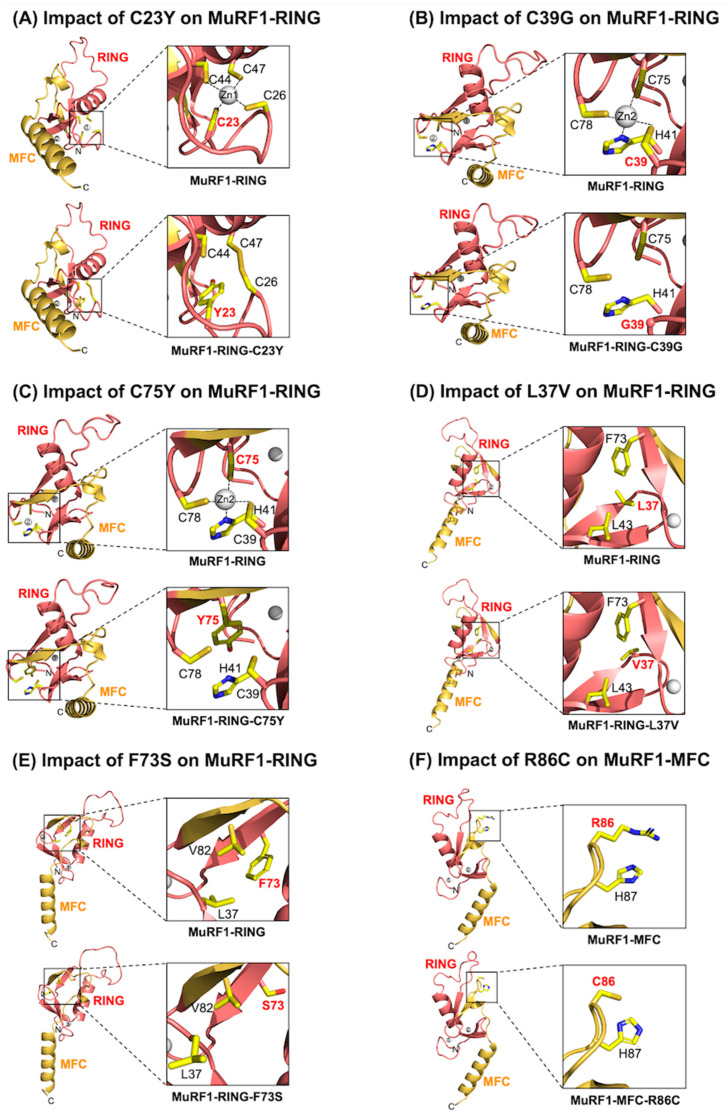
Assessing the impact of MuRF1 variants on the MuRF1-RING-MFC domain structure: Variants C23Y, C39G, and C75Y are predicted to impair zinc binding, whereas L37V, F73S, and R86C are predicted to compromise structural integrity. Ribbon representations illustrate models of the MuRF1-RING-MFC structure and its variants. (**A**) C23 coordinates the first zinc ion (top panel). The introduction of the C23Y variant is predicted to disrupt zinc binding (lower panel), (**B**) C39 coordinates the first zinc ion (top panel). The introduction of the C39G variant is anticipated to impair zinc binding (lower panel). (**C**) C75 coordinates the second zinc ion (top panel). The introduction of the C75Y variant is predicted to disrupt zinc binding (lower panel). (**D**) L37 mediates non-polar contacts with L43 and F73 (top panel). The introduction of the L37V variant is predicted to mediate fewer non-polar contacts with neighbouring residues (lower panel). (**E**) F73 mediates extensive networks of non-polar contacts with L37 and V82 (top panel). The introduction of the F73S variant is predicted to mediate fewer contacts in this region (lower panel). (**F**) R86 mediates cation–π interactions with H87 (top panel). The introduction of the R86C variant is anticipated to lead to a loss of this interaction (lower panel). Close-up views of relevant interactions are provided in boxes. The black dashed line represents zinc coordination.

Among the seven HCM-associated *MuRF1* variants located within the MuRF1-RING domain (Figure S3B), three variants (C23Y, C39G and C75Y) affected residues that are critical for coordinating a zinc ion, which is essential for preserving the structural integrity of the RING domain [28] (Figure 4A–C). In the WT structure, the highly conserved C23 is a key component of the first zinc-binding site (Figure 4A: top panel). The C23Y variant, which replaces cysteine with a bulkier tyrosine residue, may hinder zinc binding through steric hindrance, thereby limiting the special configuration necessary for effective zinc coordination (Figure 4A: lower panel). In addition, C39 in the MuRF1-RING domain contributes to the second zinc-binding site in concert with H41, C75, and C78. The C39G variant introduces a glycine residue, which lacks the capacity to coordinate zinc (Figure 4B: lower panel). Similarly, C75, also located within the second zinc-binding site, plays an integral role in stabilising the zinc ion (Figure 4C: top panel). The introduction of the C75Y variant is anticipated to exert a similar effect as C23Y by inhibiting zinc coordination (Figure 4C: lower panel). Collectively, these variants are predicted to destabilise the RING domain by compromising zinc binding, which could impair the structural integrity and functional role of MuRF1.

Three additional variants (L37V, A48V, and F73S) participate in stabilising the MuRF1-RING domain core. In the WT MuRF1-RING domain, L37 mediates multiple stabilising hydrophobic interactions with L43 and F73 (Figure 4D: top panel). The L37V variant, which substitutes leucine with smaller valine, is predicted to mediate fewer contacts with L43 and F73, leading to a destabilised core (Figure 4D: lower panel). Similarly, F73 plays a crucial role in stabilising the MuRF1-RING core region by forming non-polar interactions with V37 and V82 (Figure 4E: top panel). The F73S variant, which introduces a smaller polar side chain, is likely to considerably reduce contacts with V37 and V84, thereby weakening the MuRF1-RING-MFC core (Figure 4E: lower panel). Consequently, the L37V and F73S variants are predicted to adversely affect the folding of the RING domain, potentially compromising MuRF1 function.

However, the precise contribution of the A48V variant to disease pathogenesis remains unclear. In the WT MuRF1-RING domain, A48 engages in hydrophobic interactions with V35 and V89, which contribute to stabilising the MuRF1-RING core (Figure S4A: top panel). Unlike the L37V and F73S variants, the replacement of alanine with the slightly bulkier valine is predicted to enhance hydrophobic interactions, potentially increasing core stability (Figure S4A: lower panel).

The final MuRF1-RING variant is S61R. S61 is a solvent-exposed residue protruding from the loop region, with no significant role in stabilising the MuRF1-RING domain (Figure S4B: top panel). The substitution of serine with arginine (S61R) introduces a positively charged residue that could potentially form a cation–π interaction with Y90, which is predicted to offer additional stability to the RING domain (Figure S4B: lower panel).

### 2.5. Assessment of the MuRF1-MFC Domain: R86C May Disrupt the Structural Integrity of the MuRF1-MFC Core, Whereas R86H and I101F Are Not Predicted to Induce Structural Changes

There are three MuRF1 HCM-linked variants (R86C, R86H, and I101F) located within the MuRF1-MFC domain (Figure S3B). For the R86C variant, the native R86 residue forms a cation–π interaction with H87, which likely contributes to stabilising the MFC fold (Figure 4F: top panel). Substituting arginine with the cysteine variant (R86C) is anticipated to disrupt this interaction, potentially destabilising the structural integrity of the MuRF1-MFC domain (Figure 4F: lower panel).

In contrast, the R86H variant may facilitate π-π stacking interactions with H87, which is predicted to stabilise the MuRF1-MFC domain (Figure S4C). Regarding the I101F variant, the native I101 residue is a solvent-exposed residue protruding from the α-helix and contributes minimally to stabilising the domain (Figure S4D: top panel). The substitution of isoleucine with phenylalanine in the I101F variant has no predicted impact on the structure as similar contacts to the wild-type structure are observed, suggesting a minimal impact on the overall fold (Figure S4D: lower panel). Hence, the exact mechanism by which the R86H and I101F variants contribute to disease pathogenesis remains unclear.

### 2.6. Assessment of the MuRF1-B-box2 Domain: Variant C145Y Disrupts Zinc Binding, While I130M Compromises Core Interactions

The previously determined MuRF1-B-box2 structure (PDB ID: 3DDT) [16] was used to assess the impact of MuRF1-B-Box2 variants on the structural integrity of the domain. Three HCM-associated variants have been identified within the MuRF1-B-box2 domain: E126D, I130M, and C145Y. Molecular models of the B-box2 domain incorporating each variant were generated using AlphaFold2 [25] and subsequently compared with the wild-type crystal structure.

**Figure 5:**
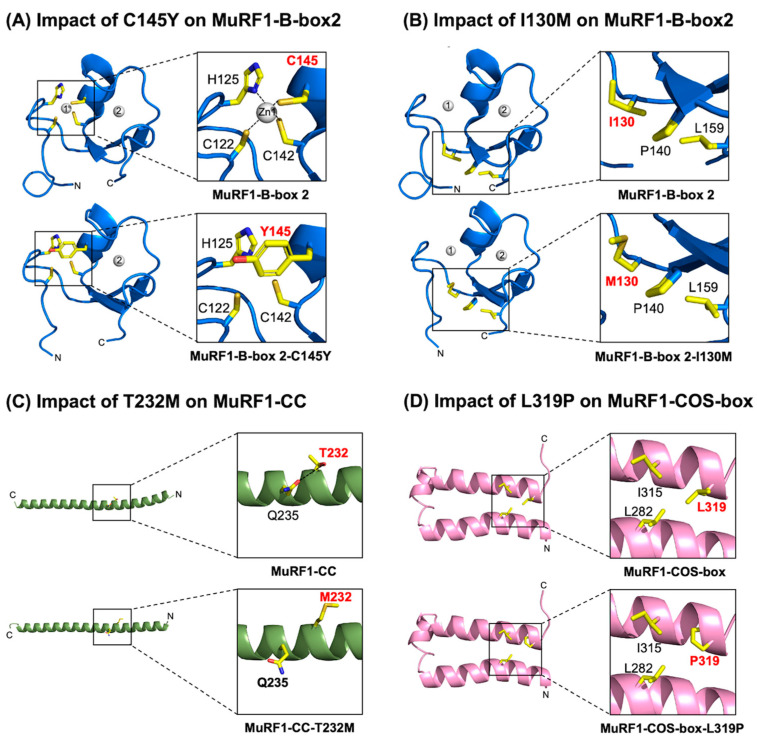
Assessing the impact of MuRF1 variants on the MuRF1-B-box2, CC, and COS-box domain structures showed that C145Y (B-box2) disrupted zinc binding, while I130M (B-box2), T232M (CC), and L319P (COS-box) weakened the core region. (**A**) Assessing the impact of the C145Y variant on the MuRF1-B-box2 domain structure. Ribbon representation of the MuRF1-B-box2 domain, highlighting that C75 coordinates the zinc ion (grey sphere). The black dashed line represents zinc binding (top panel). The introduction of the C145Y variant is predicted to disrupt zinc binding (lower panel). (**B**) Evaluating the effect of the I130M mutation on the MuRF1-B-box2 domain structure. Ribbon representation of the MuRF1-B-box2 domain, illustrating that I130 mediates non-polar contacts with P140 and L159 (top panel). The introduction of the I130M variant is predicted to decrease the number of non-polar contacts (lower panel). (**C**) Probing the effect of the T232M variant on the MuRF1-CC domain structure. Ribbon representation of the MuRF1-CC domain model, showcasing that T232 mediates a hydrogen bond with Q235. The black dashed line represents the hydrogen bond (top panel). The introduction of the T232M variant is predicted to disrupt this interaction (lower panel). (**D**) Probing the impact of the L319P variant on the MuRF1-COS-box domain structure. Ribbon representation of the MuRF1-COS-box domain model, showing that L319 mediates multiple hydrophobic interactions with L282 and I315 (top panel). The introduction of the L319P variant is predicted to reduce these interactions (lower panel). Close-up views of relevant interactions are provided in boxes.

In the WT MuRF1-B-box2 domain, the highly conserved C145 residue is a crucial component of the first zinc-binding site. Along with C122, H125, and C142, it coordinates the first zinc ion, which is essential for maintaining the correct conformation of the B-box2 domain (Figure 5A: top panel). Replacing cysteine with a bulkier tyrosine residue (C145Y) is predicted to disrupt this zinc coordination, thereby impeding effective zinc binding and compromising the structural integrity of the B-box domain, which could impair MuRF1 function (Figure 5A: lower panel).

For the I30M variant, the native I130 residue engages in multiple non-polar interactions with P140 and L159, contributing to stabilising the B-box2 domain core (Figure 5B: top panel). The introduction of methionine at this position is anticipated to mediate fewer hydrophobic interactions with P140, potentially leading to reduced stability of the B-box 2 core (Figure 5B: lower panel).

In contrast, the pathogenic contribution of the E126D variant is less well defined. The native surface-exposed E126 residue protrudes from the loop region without significantly contributing to stabilising the domain (Figure S5: top panel). The replacement of glutamate with aspartate preserves the negative charge and is predicted to not have an impact on the overall B-Box2 domain fold (Figure S5: lower panel). However, given that the B-box2 and COS-box domains represent flanking motifs crucial for oligomerisation and maintaining the quaternary structure of MuRF1 [29], it is plausible that the E126D variant could adversely affect this arrangement, potentially influencing the functional dynamics of MuRF1.

### 2.7. Assessment of the MuRF1-CC Domain: The T232M Variant Potentially Destabilises the α-Helical Structure

We initially considered using the previously determined crystal structure of MuRF1-CC (PDB ID: 4M3L) [17] to evaluate whether the identified variants compromise the structural integrity of the CC domain. The native structure features two parallel dimers arranged in an open-scissor configuration. However, this parallel tetrameric arrangement is unlikely to represent the physiologically relevant state of MuRF1-CC, as previous studies employing SEC-MALS (size exclusion chromatography with multi-angle static light scattering) [17], disulfide cross-linking [29], electron paramagnetic resonance (EPR), and pulsed electron–electron double resonance (PELDOR) approaches [29] suggest that the MuRF1-CC domain forms an antiparallel homodimer. Consequently, an anti-parallel dimer model of MuRF1-CC was generated by superimposing the MuRF1-CC domain onto the anti-parallel TRIM25-HD (helical domain of TRIM25) homodimer (PDB ID: 4LTB) [30] prior to assessing the impact of CC variants.

There are two HCM-linked variants within the MuRF1-CC domain (T232M and D254N). Following the mapping of these variants onto the MuRF1-CC anti-parallel dimer model, it is evident that they are unlikely to disrupt the assembly of the dimer (Figure S6A). Consequently, alternative mechanisms were considered regarding their contribution to disease pathology. One possibility was that these mutations could compromise the structural integrity of the MuRF1-CC domain and contribute to the pathophysiology of HCM. To explore this hypothesis, models of the MuRF1-CC domain incorporating each variant were generated using AlphaFold2 [25]. Importantly, the variant models exhibited a strong alignment with the MuRF1-CC crystal structure, indicating that the overall structural framework remains intact.

In the WT MuRF1-CC domain, the polar T232 residue plays a significant role in stabilising the CC domain by forming a hydrogen bond with Q235 (Figure 5C: top panel). Replacing threonine with the methionine variant (T232M) introduces a larger hydrophobic side chain in proximity to the polar Q235 residue. This alteration is predicted to disrupt this hydrogen bond, potentially weakening the α-helical structure within the MuRF1-CC domain (Figure 5C: lower panel).

Conversely, in the case of the D254N variant, the implications for pathogenic action are less straightforward. The native D254 residue forms a salt bridge with K258 (Figure S6B: top panel). The replacement of aspartate with the asparagine variant (D254N) introduces an uncharged polar side chain, which is predicted to form a compensatory hydrogen bond with K258, thereby stabilising the region (Figure S6B: lower panel). This observation suggests that the D254N variant is not predicted to have an impact on the structure of the CC domain, and other mechanisms may contribute to the pathophysiology of HCM.

### 2.8. Assessment of the MuRF1-COS-Box Domain: Variant L319P May Compromise MuRF1’s Structural Integrity

In the absence of an experimentally derived structure of the MuRF1-COS-box domain, a molecular model was generated using Phyre2 [31]. The resulting MuRF1-COS-box model demonstrated a high degree of structural alignment with the MID1 (TRIM18)-COS-box domain (PDB ID: 5IM8) [32], yielding an RMSD of 0.468 Å. This suggested that the model could reliably be employed for comparative analyses with MuRF1-COS-box structures that encompass disease-associated variants.

Four HCM-linked variants (M305I, A318D, L319P, and A321D) have been identified within the MuRF1-COS-box domain. The L319P variant is particularly noteworthy; the native leucine residue mediates multiple hydrophobic interactions with L282 and I315, thereby stabilising the core region (Figure 5D: top panel). Replacing leucine with the proline variant (L319P) introduces a smaller non-polar side chain, which is predicted to reduce these interactions, and potentially destabilizes the domain (Figure 5D: lower panel). Such alterations could adversely affect the conformation of the COS-box domain and compromise MuRF1 function.

In contrast, the M305I and A321D variants are predicted to have no impact on the domain structure. The native M305 residue forms a non-polar interaction with the aliphatic regions of S290, contributing to stabilising the COS-box domain core (Figure S7A: top panel). Substituting methionine with the isoleucine variant (M305I) preserves interactions with S290 and is predicted to maintain the stability of the core domain (Figure S7A: lower panel).

Similarly, the A321D variant is predicted to have no impact on the structure either. A321 is a surface-exposed residue that plays no significant role in COS-box domain stability (Figure S7C: top panel). The substitution of alanine with aspartate (A321D) introduces a negatively charged moiety which appears to not adversely impact the stability of the COS-box domain (Figure S7C: lower panel).

Finally, with respect to the A318D variant, the native A318 residue again plays no significant role in stabilising the domain (Figure S7B: top panel). Nevertheless, replacing alanine with an aspartate residue (A318D) is predicted to potentially introduce a hydrogen bond with H314, which may enhance the stability of the COS-box domain (Figure S7B: lower panel).

It is possible that these variants may affect MuRF1 function via other mechanisms within cellular processes, such as alterations in localisation, protein–protein interactions, or post-translational modifications, thereby contributing to disease pathogenesis.

## 3. Discussion

MuRF1 (Muscle RING-finger protein-1, coded for by the gene *TRIM63*) is a crucial E3 ubiquitin ligase that plays a significant role in controlling cardiac cell size and function [12]. Multiple clinical studies have identified that genetic variants of *TRIM63* are associated with HCM [9,19,20,21,22,23,24,33], a disease characterised by the thickening of the heart muscle. Therefore, *TRIM63* is included in clinical genetic testing panels for HCM, such as those provided by Genomics England [34] and by Mayo Clinic [35].

So far, only three variants of MuRF1 (A48V, I130M, and Q247*) have been investigated for their pathophysiological effects. Using transgenic mouse hearts, experiments indicated that these three variants caused cardiac hypertrophy by enhancing hypertrophic signalling and markers [19].

Thus, elucidating the mechanisms by which MuRF1 variants contribute to the pathogenesis of HCM is essential for developing novel targeted therapeutic strategies. This study examined the impact of 25 HCM-associated MuRF1 variants on ubiquitylation using in vitro ubiquitylation assays and on protein structure with computational prediction approaches.

In our in vitro ubiquitylation experiments, we evaluated the impact of MuRF1 variants on the ubiquitylation process, specifically focusing on monoubiquitylation. Our experiments revealed that 17 out of 25 HCM-associated MuRF1 variants, to varying extents, decreased the auto-monoubiquitylation (Auto-mUb) of MuRF1, indicating a potential impairment of its function. Notably, most of these variants mapped to the RING, B-Box2, and COS-box domains, underscoring the functional importance of these domains in the Auto-mUb process. Conversely, among the eight MuRF1 variants exhibiting Auto-mUb activity levels comparable to the wild type, four were located within the MFC (R86H and I101F) and CC (T232M and D254N) domains, suggesting that these modules may exert a minimal impact on Auto-mUb and play less significant functional roles.

We further extended our study to evaluate the effects of these variants on the monoubiquitylation of the MuRF1 substrate, titin A168-A170 (His-Titin-mUb). Remarkably, all 25 HCM-linked MuRF1 variants tested compromised His-Titin-mUb. Among these, 16 variants completely abolished His-Titin-mUb activity, with the majority again mapping to the RING (C23Y, L37V, C39G, A48V, S61R, and C75Y), B-box2 (I130M, C145Y, and S161Cfs*8), and COS-box (A318D, L319P, A321D, and E299*) domains. This observation highlights the critical roles of these domains in His-Titin-mUb and suggests that MuRF1 variants exhibit a heightened sensitivity in affecting substrate ubiquitylation compared to their impact on MuRF1 auto-ubiquitylation. The extensive binding interface between MuRF1 and titin (A168-A170), which encompasses multiple domains, including the RING, MFC, B-box2, CC, and COS-box domains [5,36], may account for this sensitivity.

The N-terminal variant S5L was found to impair Auto-mUb, likely due to UBE2W preferentially attaching ubiquitin to the N-terminus of proteins rather than lysine residues [37]. Additionally, the S5L variant disrupted His-Titin-mUb, potentially because this residue is crucial for maintaining a MuRF1 conformation essential for substrate ubiquitylation. This is reminiscent of the role played by the N-terminal residue E13 of TRIM21, which stabilises the closed conformation of TRIM21-RING in a complex with ubiquitin-conjugated UBE2W. This interaction is mediated by ubiquitin residue K11 and facilitates ubiquitylation in a UBE2W-dependent manner [26]. However, the precise mechanism by which the MuRF1 S5L variant contributes to HCM pathogenesis remains undefined.

Similarly, the AT variant G351R exhibited negative effects on both Auto-mUb and His-Titin-mUb, potentially due to its location within the intrinsically disordered region, which is crucial for the ubiquitin–proteasome system as it mediates protein–protein interactions and the recognition of target proteins [38]. Nevertheless, this explanation does not comprehensively clarify the role of the MuRF1-G351R variant in HCM pathogenesis. To gain deeper insights into the involvement of these MuRF1 variants in HCM, further studies focusing on the underlying pathogenic mechanisms are warranted, using cardiomyocytes or in vivo settings.

The four truncated variants of MuRF1 are located in distinct domains: B-box2 (K146Tfs*24 and S161Cfs*8), CC (Q247*), and COS-box (E299*). These truncated variants impaired both Auto-mUb and His-Titin-mUb, with E299* demonstrating the most pronounced impact by abolishing MuRF1 function in both processes. Truncation variants frequently trigger the nonsense-mediated decay (NMD) pathway, which degrades mRNA with premature termination codons to prevent the synthesis of faulty proteins. As a result, they not only yield truncated proteins but may also reduce the amount of proteins [39]. Interestingly, truncation variants can manifest milder disease phenotypes than missense variants if NMD activation inhibits protein synthesis, thereby preserving one wild-type allele to maintain normal protein function [40]. However, our findings demonstrate that the four truncated MuRF1 variants, which were recombinant proteins constructed in bacteria, exerted a more significant negative impact on MuRF1 ubiquitylation compared to missense variants. Consistent with this, a previous study reported that the MuRF1-Q247* truncation variant exhibited stronger adverse effects on MuRF1 activities in adult cardiomyocytes and mice hearts, compared to missense variants A48V and I130M [19]. This suggests that these truncation variants are likely to be dominant negative.

The variants in this study were expressed in bacteria lacking the NMD pathway, and in vitro ubiquitylation assays alone are insufficient to fully elucidate the role of these truncated MuRF1 variants in HCM pathogenesis. Thus, further experiments are essential to clarify the mechanisms by which truncated MuRF1 variants contribute to HCM pathogenesis. For instance, it is of interest to elucidate whether truncated MuRF1 variants could be detected in human HCM hearts using a top-down proteomics method. This approach enables the detection of proteoforms, distinct forms of a protein arising from transcription and translation, and the analysis of post-translational modifications of truncated variants without fragmenting them into smaller peptides [41].

The MuRF1-RING domain appears to be the most sensitive region, as variants within this domain resulted in significant impairment of both MuRF1 auto-monoubiquitylation and titin monoubiquitylation. This finding is not surprising given the critical role of the RING domain in mediating UBE2W binding, which is essential for MuRF1 autoubiquitylation. Intriguingly, based on our structural modelling predictions, none of the MuRF1-RING domain variants directly affected the putative UBE2W-binding motif. Instead, most of these variants were predicted either to disrupt zinc coordination (C23Y, C39G, and C75Y) or to compromise critical core interactions (L37V and F73S), rationalising their detrimental impact on MuRF1 ubiquitylation function. This observation aligns with a previous study demonstrating the complete inhibition of MuRF1 E3 ligase activity following the mutation of cysteine residues (C44 and C47) that coordinate the first zinc-binding site [42]. Moreover, the ubiquitylation function of a related E3 ubiquitin ligase, c-Cbl protein, was compromised following the removal of the RING domain or the first zinc-binding site of the RING domain [43,44]. In contrast, the disease contributions of the A48V and S61R variants were less straightforward, as our structural modelling predicted that these changes would strengthen interactions despite both variants abolishing His-Titin-mUb. Further investigation will be necessary to elucidate how these variants contribute to the pathogenesis of HCM.

Variants mapping to the B-box2 domain, namely C145Y and I130M, impaired MuRF1 Auto-mUb and His-Titin-mUb. Our structural predictions indicate that C145Y is predicted to disrupt zinc coordination, while I130M is predicted to compromise core-stabilising interactions, potentially destabilising the B-box2 fold. These structural alterations provide a plausible explanation for how these variants impact MuRF1 ubiquitylation function and contribute to HCM pathogenesis. The contribution of the E126D variant to disease pathogenesis is less clear. In vitro ubiquitylation assays revealed a minimal impact on MuRF1 Auto-mUb, and while His-Titin-mUb was affected, it belonged to a group that was less impacted compared to other variants. Consistent with these results, our structural modelling predictions suggest that the E126D substitution has no impact on the structure and likely preserves the integrity of the B-Box2 domain.

Among the COS-box domain variants, all four (M305I, A318D, L319P, and A321D) impaired His-Titin-mUb, but only two (A318D and L319P) compromised MuRF1 Auto-mUb. The impact of the L319P variant on MuRF1 structure can be rationalised, as this change is predicted to disrupt core-stabilising interactions, potentially compromising the stability of the COS-box domain. In contrast, the M305I variant, which was the least sensitive to impairing ubiquitylation among the COS-box variants, was predicted to have no structural impact. The disease contributions of the A318D and A321D variants are less well understood, as our structural predictions suggest that these changes exert no impact on the structure or could potentially strengthen interactions, despite their impact on MuRF1 ubiquitylation functionality.

MuRF1 variants mapped to the MFC and CC domains exhibited the least impairment in MuRF1 Auto-mUb and His-Titin-mUb. Structural modelling predicted that the R86H and I101F variants had no impact on the structure and preserved the overall integrity of the MFC domain. For the R86C variant, which impacted both MuRF1 Auto-mUb and His-Titin-mUb, our predictions suggest that this change reduces key core-stabilising contacts, potentially impairing MFC domain stability. In the CC domain, the T232M and D254N variants exerted no impact on Auto-mUb but affected His-Titin-mUb, albeit to a lesser extent, compared to other MuRF1 variants. Our structural predictions indicate that the T232M variant may destabilise the α-helical structure, whereas the D254N variant is predicted not to impact the structure, leaving the integrity of the CC domain largely unaffected.

The mechanisms by which variants A48V, S61R, and A318D lead to disease pathogenesis remain undefined. These variants are predicted to strengthen interactions within their respective domains, yet based on our in vitro functional assays, they completely abolish titin ubiquitylation. One plausible explanation is that increased interactions could reduce the flexibility of these domains, thereby disrupting substrate binding and impairing MuRF1 ubiquitylation activity. The flexibility of MuRF1 domains is critical for optimal substrate recognition and catalytic function, so these variants may compromise MuRF1 protein dynamics [45,46].

Equally confounding is the observation that some variants (R86H, I101F, E126D, D254N, M305I, and A321D) are predicted to have no structural impact despite exhibiting reduced functionality in our in vitro ubiquitylation assay using the His-Titin-mUb substrate. The lack of predicted structural alterations in these six HCM-linked MuRF1 variants does not necessarily preclude their involvement in disease pathogenesis. Previous studies have demonstrated that MuRF1 requires dimerisation or high-order oligomerisation to effectively ubiquitylate titin [5,26,47]. The dimerisation of the RING domain is crucial for the ubiquitylation of a neighbouring RING monomer at the N-terminal [26,47]. Furthermore, some RING domain-containing proteins require even higher-order oligomerisation, such as tetramerisation, to ubiquitylate their cognate substrates [26]. Therefore, it is conceivable that these variants may affect the ability of MuRF1 to dimerise or oligomerise. This disruption could impair the ubiquitylation of titin, which relies on a specific MuRF1 dimeric state for efficient substrate recognition and modification [5,26,47]. In contrast, the MuRF1 monomer can be ubiquitylated by the MuRF1 dimer. Therefore, the auto-ubiquitylation of MuRF1 may be less sensitive to changes in its oligomeric state, rendering it less susceptible to such disruptions. Supporting this idea, isothermal titration calorimetry studies have identified the MuRF1 region spanning residues G165-I315 as crucial for titin binding [5], and some of these ‘structurally neutral’ variants fall within this region.

Other potential mechanisms may also explain the apparent disconnect between the lack of structural effects and reduced MuRF1 functionality. These variants could interfere with post-translational modifications that regulate MuRF1 activity or affect intracellular trafficking. Further experiments are needed to determine which of these mechanisms contribute to the observed functional deficits.

In conclusion, the structural analysis of HCM-associated MuRF1 variants highlights the importance of considering not only the direct structural consequences of these variants but also their potential impact on the oligomerisation and functional properties of the protein. Further investigations into the oligomeric state of MuRF1 and its relationship with substrate ubiquitylation will be crucial for understanding the molecular mechanisms underlying the pathogenesis of HCM-associated MuRF1 variants.

Although structural modelling provides valuable insights into the structural impacts of variants, it has inherent limitations. Specifically, this prediction approach may struggle to evaluate broader topological effects or to accurately model domain–domain orientations for multi-domain proteins like MuRF1. This highlights the importance of understanding how MuRF1 variants predicted to be structurally neutral may contribute to disease pathogenesis. To gain a more comprehensive understanding of the pathogenesis of MuRF1-related HCM, future work should focus on determining the full-length three-dimensional structure of MuRF1 and its variants using X-ray crystallography and cryo-electron microscopy approaches to experimentally characterise the impact of variants on protein structure and function. NMR spectroscopy could also be utilised to assess how HCM-associated variants in MuRF1 influence its structural dynamics and conformational stability in solution. Additionally, obtaining MuRF1 crystal structures in complex with its key partners, such as UBE2W, monoubiquitin, and the titin A168-A170 region, will be crucial for investigating the impact of MuRF1 variants on protein dynamics and interactions. Additionally, binding assays such as isothermal titration calorimetry or co-immunoprecipitation could be used to assess the impact of MuRF1 variants on interactions with key partners, including UBE2W, Titin A168-A170, and monoubiquitin. These approaches would provide both quantitative and qualitative insights into how variant-induced changes influence binding affinity and complex formation, thereby elucidating the underlying molecular mechanisms.

It is also important to note that the in vitro experiments conducted in this study do not fully reflect the complex activities of MuRF1 within the heart. For example, the bacterially expressed proteins used in the experiments do not carry post-translational modifications, such as phosphorylation, which may play an important role in regulating MuRF1 function. This limitation may apply to those variants that are predicted to either enhance interactions or to have no impact on the structure. To more accurately capture the pathogenic actions of HCM-associated MuRF1 variants, future investigations should employ cellular models, such as human induced pluripotent stem cell-derived cardiomyocyte models (iPSC-CMs) [48] or in vivo models. For the latter, studies in mice and zebrafish are commonly used [49,50]. These approaches can better replicate the pathological mechanisms of MuRF1 variants, providing valuable mechanistic insights into heart function.

Overall, this comprehensive analysis of the functional and structural consequences of MuRF1 variants provides valuable insights into the mechanisms by which specific variants contribute to HCM pathogenesis (Table 1). Further investigation will be necessary to fully elucidate the complex interplay between structural alterations, ubiquitylation dynamics, and disease development.

## 4. Materials and Methods

### 4.1. PCR Mutagenesis

The plasmid pMEX3Cb-MBP-TEV-TRIM63 served as a template for generating 25 *TRIM63* variants, encompassing missense, nonsense, and frameshift mutations, through one-step site-directed plasmid mutagenesis [51]. Primers for the 25 *TRIM63* variants were designed in accordance with the modified site-directed plasmid mutagenesis protocol [51], using the Benchling platform (https://www.benchling.com, accessed on 1 March 2022–30 April 2023) and NEB Tm Calculator software (https://tmcalculator.neb.com/#!/main, accessed on 1–31 January 2022) (detail in Table S4). The details of the primer sequences are presented in Table S3. The PCR reaction was performed in 25 μL comprising 1 μL of 1 ng/μL plasmid template, 1.25 μL of 10 μM forward primer, 1.25 μL of 10 μM reverse primer, 0.5 μL of 10 mM dNTP (Deoxynucleoside triphosphate) (New England Biolabs, N0447S, Ipswich, MA, USA), 10.75 μL of Nuclease-free Water (New England Biolabs, B1500S, Ipswich, MA, USA), 5 μL of 5× Q5 Reaction buffer, 5 μL of 5× Q5 High GC Enhancer, and 0.25 μL of Q5 high-fidelity DNA polymerase (New England Biolabs, M0491S, Ipswich, MA, USA). The PCR amplification procedure consisted of 35 cycles, which was initiated with a denaturing step at 98 °C for 10 s, followed by an annealing step at 67 °C for 30 s, and concluded with an extension step at 72 °C for 3 min and 30 s. Next, the reaction mixture was treated with 0.5 μL of DpnI (New England Biolabs, R0176S, Ipswich, MA, USA) and incubated at 37 °C for 2 h, followed by a heat incubation step at 80 °C for 20 min to selectively digest *E. coli*-derived DNA, thereby isolating the PCR-synthesised DNA (PCR product). The resulting PCR product was transformed into DH5α competent cells to insert the mutated plasmid into *E. coli.* A single colony was selected for the propagation and purification of the mutated plasmid using the Mini-prep method. The accuracy of the Mini-prep product or mutated plasmid was confirmed via DNA sequencing using the Sanger sequencing portal provided by Source BioScience service (Nottingham, UK).

### 4.2. Protein Expression and Purification

Both mutated MBP-MuRF1 and WT MBP-MuRF1 plasmids were transformed into BL21(DE3) competent *E. coli* cells. A single colony was selected for mini-inoculation (4 mL) and overnight (50 mL) inoculation in LB broth media (Merk, 1102855000, Darmstadt, Germany) supplemented with 100 μg/mL of ampicillin (Merk, A9518-5G, Darmstadt, Germany). (Mini-inoculation was used as a primary culture for overnight inoculation.) Next, the 50 mL culture was scaled up to 1 L in LB media containing 100 μg/mL ampicillin and 200 μM ZnSO_4_ (Merk, 7446-20-0, Darmstadt, Germany) and incubated at 37 °C with shaking at 180 rpm. Once the culture reached an OD 600 (optical density at 600 nm) of approximately 0.5, the temperature was lowered to 18 °C. The induction of protein expression was initiated through the addition of isopropyl β-D-1-thiogalactopyranoside (IPTG) (Thermo Fisher Scientific, 367-93-1, Waltham, MA, USA) when the OD 600 reached between 0.6 and 0.7, achieving an IPTG final concentration of 250 μM, and the culture was incubated overnight under the same conditions. For the expression of His-UBE2W, the transformation and protein expression protocols were similar to MBP-MuRF1, with the exception that 50 μg/mL kanamycin (Merk, 60615, Darmstadt, Germany) was used, with higher IPTG concentration (0.5 mM), without the addition of ZnSO_4_.

The cultures (mutated MBP-MuRF1, WT MBP-MuRF1, and His-UBE2W cultures) were harvested via centrifugation at 4200 rpm for 15 min at 4 °C. The resulting pellets were resuspended in the appropriate lysis buffers: the MBP-tag buffer comprised 50 mM Tris-HCl (pH 7.5) (Merk, T5941, Darmstadt, Germany), 150 mM NaCl (Merk, 106404, Darmstadt, Germany), 5% Glycerol (Merk, G7893, Darmstadt, Germany), 1 mM TCEP (Tris-(2-Carboxyethyl)phosphine, Hydrochloride) (Thermo Fisher Scientific, 77720, Waltham, MA, USA), and 1 mM PMSF (Phenylmethylsulfonyl fluoride) (Thermo Fisher Scientific, 329-98-6, Waltham, MA, USA), while the His-tag buffer consisted of 50 mM Tris-HCl pH 8.0, 150 mM NaCl, 50 mM Imidazole (Merk, 288-32-4, Darmstadt, Germany), 0.5 mM TCEP, and 1 mM PMSF. Cell lysis was performed using the Emulsiflex C3 Cell Disruptor (Avestin Europe, Mannheim, Germany). Cells lysate was centrifuged at 5000 rpm for 2 h at 4 °C, followed by filtration through a 0.45 μm syringe filter (Merk, SLHAR33SS, Darmstadt, Germany) to remove cellular debris. Recombinant WT MuRF1 and mutant proteins were purified using amylose resin (New England Biolabs, Ipswich, MA, USA), while His-UBE2W was purified via a His-Trap column (GE Healthcare, Chicago, IL, USA). The purification protocols adhered to the manufacturers’ instructions. To assess protein purity, PageBlue Protein Staining Solution (Thermo Fisher Scientific, 24620, Waltham, MA, USA) was used in accordance with the manufacturer’s protocol. Protein concentration was quantified using an LVis Plate (BMG LABTECH, Ortenberg, Germany) equipped with 16 micro-drop wells. Finally, the purified recombinant protein samples were stored at −80 °C for the subsequent experiments.

### 4.3. In Vitro Ubiquitylation

Immunoblotting confirmed equal loading and the stability of the 25 MuRF1 variants prior to conducting in vitro ubiquitylation experiments (Figure S8).

The in vitro ubiquitylation reactions were conducted in a tube containing 50 mM HEPES pH 7.5 (Merk, 391340, Darmstadt, Germany), 1 mM DTT (1,4-dithiothreitol) (Merk, 3483-12-3, Darmstadt, Germany), 10 mM MgCl_2_ (Merk, 7786-30-3, Darmstadt, Germany), 1 mM ATP (adenosine triphosphate) (Merk, 34369-07-8, Darmstadt, Germany) pH 7.0, 0.04 μg/μL ubiquitin, 0.004 μg/μL HIS-UBE1, 0.012 μg/μL UBE2W, and 0.05 μg/μL of either WT or mutated MBP-MuRF1. For titin ubiquitylation, a His-Titin fragment (A168-A170) was incorporated into the reaction mixture at a concentration of 0.01 μg/μL. Both auto-ubiquitylation and titin ubiquitylation reactions were incubated at 37 °C, with agitation at 1000 rpm for 1 h using the Thermoshaker (Eppendorf, Hamburg, Germany). The reactions were terminated via the addition of a buffer containing 1X NuPAGE LDS Sample Buffer (4X) (Thermo Fisher Scientific, NP0008) and 1.25% β-Mercaptoethanol (VWR, 60-24-2, Radnor, PA, USA). Subsequently, the samples were allowed to incubate at room temperature overnight to facilitate denaturation prior to immunoblotting.

### 4.4. Immunoblotting

For immunoblotting, samples were loaded onto 8% or 10% Bis-Tris gels and subjected to sodium dodecyl sulphate–polyacrylamide gel electrophoresis (SDS-PAGE) to separate proteins based on their molecular weight. Gels were run in 1× MOPS buffer, employing a two-step voltage protocol: 100 volts for 10 min followed by 140 volts for approximately 90 min. Following electrophoresis, proteins were transferred to 0.2 μm polyvinylidene fluoride (PVDF) membranes (Cytiva, 10600021, Marlborough, MA, USA) at 100 volts for 1 h. Membranes were blocked in 5% milk diluted in 1xTris-buffered saline–Tween 20 (TBS-T) for 1 h. After blocking, membranes were rinsed three times with 1xTBS-T and incubated overnight with the appropriate primary antibodies: Anti-MBP (HRP) (1:20,000, New England Biolabs, E8038S, Ipswich, MA, USA), Anti-6xHis (1:10,000, Thermo Fisher Scientific, Clontech-631212, Waltham, MA, USA), and Anti-FK2 (1:1000, Merk, 04-263, Darmstadt, Germany). Following primary antibody incubation, membranes were washed three times with 1xTBS-T for 10 min before a 1 h incubation at room temperature with horseradish peroxidase (HRP)-conjugated secondary antibodies. Membranes were rewashed three times with 1xTBS-T prior to antibody detection using an enhanced chemiluminescent HRP substrate detection kit (Merck, WBKLS0500, Darmstadt, Germany). Imaging of blots was performed using G:BOX Chemi-XR5 (Syngene, Cambridgeshire, UK).

### 4.5. Statistical Analysis

Statistical analysis was performed using the GraphPad Prism 10.0.0 software. The data pertaining to ubiquitylation activity were presented as the mean ± standard error of the mean (SEM), with biological replicates (n = 3) presented as fold changes relative to the wild type (WT). One-way ANOVA followed by Dunnett’s test was employed to assess statistical significance, with *p*-values < 0.05 considered indicative of statistical significance, as denoted by the following hash signs (#): ^#^ *p* < 0.05,^##^ *p* < 0.01, ^###^ *p* < 0.001, ^####^ *p* < 0.0001, compared to WT.

### 4.6. Structure Modelling of MuRF1-RING-MFC, MuRF1-COS-Box, and the MuRF1/UBE2W Complex

Due to the lack of experimentally derived structure for the MuRF1-RING-MFC and MuRF1-COS-box domains, molecular models were generated using computational modelling approaches, specifically AlphaFold2 and Phyre2. Briefly, the MuRF1 sequences corresponding to the RING-MFC (residues M1-P113) and COS-box (residues G272-E328) domains were retrieved from UniProt accession number Q969Q1 and submitted to AlphaFold2 for RING-MFC domain modelling and Phyre2 for COS-box domain modelling (Table S4). The resulting models of MuRF1-RING-MFC and MuRF1-COS-box were superimposed onto existing homologue structures: TRIM21-RING (PDB ID: 8A58) and MID1-COS-box (PDB ID: 5IM8) to validate the reliability of the models. Furthermore, the MuRF1-RING-MFC model was superimposed onto the TRIM-21/UBE2W complex crystal structure (PDB ID: 8A58) to generate a preliminary model of the MuRF1-RING-MFC/UBE2W complex. However, this initial crude model exhibited steric clashes, necessitating further refinement through docking simulations with the HADDOCK programme (Table S4).

### 4.7. Evaluating the Impact of MuRF1 Variants on MuRF1 Structure

The structural effects of 19 MuRF1 variants were evaluated using AlphaFold2 and Phyre2 to predict their three-dimensional structures. Variant models were superimposed onto the wild-type (WT) structure, and molecular interactions around the variant sites were analysed using PyMOL Molecular Graphics System version 2.6.0 (Table S4) to identify structural alterations. The structural roles of the WT residues were assessed to predict how substitutions might affect MuRF1 structure, focusing on disruptions to UBE2W ligand binding, zinc coordination, and domain integrity. The process involved comparing WT-mediated molecular interactions to those in variant models to pinpoint structural changes.

## 5. Conclusions

This study has shed light on the molecular mechanisms of HCM-associated MuRF1 variants. It revealed that all 25 MuRF1 variants tested may impair ubiquitylation. Ten are predicted to destabilise the protein’s structure (Table 1). All 25 variants investigated negatively affected its ability to monoubiquitylate MuRF1’s substrate (titin A168-A170), while 17 variants impaired or completely abolished auto-monoubiquitylation. Structural modelling predictions of 19 variants proposed the disruption of zinc binding and the compromise or strengthening of the structural integrity as underlying explanations, while domain stabilisation may impair MuRF1 function for six variants (R86H, I101F, E126D, D254N, M305I, and A321D). The fact the structural predictions could not identify a molecular explanation for reduced ubiquitylation function on the six variants points at the characteristics of MuRF1, which were not considered with structural modelling (e.g., dimerisation, oligomerisation, or its behaviour in multi-protein complexes). Future work will investigate the impact of the MuRF1 variants in cardiac, cellular, or in vivo systems. Moreover, innovative structural biology approaches are needed to elucidate the structure of native full-length MuRF1 in complex with UBE2W, monoubiquitin, and its substrates, including itself and titin A168-170. These approaches will provide insights into the protein conformation of MuRF1 during its interaction with cognate partners or substrates, which can be utilised for disease prognosis and drug discovery.

## Figures and Tables

**Table 1:** Summary of the in vitro ubiquitylation assay results, including MuRF1 auto-monoubiquitylation (Auto-mUb) and titin (A168-A170) monoubiquitylation (His-Titin-mUB) alongside protein structure prediction data for each MuRF1-associated HCM variant. The degree of decreased ubiquitylation is shown with a hash symbol (#): ^#^ *p* < 0.05, ^##^ *p* < 0.01, ^###^ *p* < 0.001, ^####^ *p* < 0.0001. The results are compared to the WT via one-way ANOVA followed by Dunnett’s test.

MuRF1 Variants	Domain	Result Summary of This Study		
		Auto-mUb	His-Titin-mUb	Structural Modelling Predicted Impact on MuRF1 Structure
S5L [9]	N-terminal	Decreased ^#^	Decreased ^####^	No prediction
C23Y [23]	RING	Abolished ^###^	Abolished ^####^	Impair zinc binding (site 1)
L37V [23]	RING	Decreased ^##^	Abolished ^####^	Compromise RING domain core region
C39G [24]	RING	Decreased ^###^	Abolished ^####^	Impair zinc binding (site 1)
A48V [19]	RING	Decreased ^##^	Abolished ^####^	Strengthen RING domain core region
S61R [9]	RING	Decreased ^#^	Abolished ^####^	Strengthen RING domain core region
F73S [9]	RING	No change	Decreased ^####^	Compromise RING domain core region
C75Y [23,24]	RING	Decreased ^#^	Abolished ^####^	Impair zinc binding (site 2)
R86C [9]	MFC	Decreased ^#^	Abolished ^####^	Compromise MFC domain stability
R86H [9]	MFC	No change	Decreased ^####^	No change; structurally neutral
I101F [9]	MFC	No change	Decreased ^####^	No change; structurally neutral
E126D [9]	B-box2	No change	Decreased ^####^	No change; structurally neutral
I130M [19]	B-box2	Decreased ^##^	Abolished ^####^	Compromise B-box2 domain core region
C145Y [23]	B-box2	Decreased ^#^	Abolished ^####^	Impair zinc binding (site 1)
K146Tfs*24 [23]	B-box2	Decreased ^####^	Decreased ^####^	No prediction
S161Cfs*8 [23,24]	B-box2	Decreased ^####^	Abolished ^####^	No prediction
T232M [9]	CC	No change	Decreased ^#^	Destabilise CC domain
Q247* [19,20,21,22,23,24]	CC	Decreased ^####^	Abolished ^####^	No prediction
D254N [9]	CC	No change	Decreased ^#^	No change; structurally neutral
E299* [9]	COS-box	Abolished ^####^	Abolished ^####^	No prediction
M305I [9]	COS-box	No change	Decreased ^####^	No change; structurally neutral
A318D [9]	COS-box	Decreased ^###^	Abolished ^####^	Strengthen COS-box domain stability
L319P [23]	COS-box	Decrease ^###^	Abolished ^####^	Compromise COS-box core region
A321D [9]	COS-box	No change	Abolished ^####^	No change; structurally neutral
G351R [9]	AT	Decreased ^#^	Abolished ^####^	No prediction

## Data Availability

The data presented in this study are included within the article. Additional queries can be addressed to the corresponding authors.

## Supplementary Materials

The supporting information can be downloaded at https://www.mdpi.com/article/10.3390/ijms26083921/s1.

## Abbreviations

The following abbreviations are used in this manuscript:

ANP	Atrial Natriuretic Peptide
AT	Acidic tail
Anti-6xHis	Anti-6xHistidines Antibody
Anti-FK2	Anti-Ubiquitinylated proteins Antibody, clone FK2
Anti-MBP (HRP)	Anti-MBP Monoclonal Antibody, HRP conjugated
ATP	Adenosine triphosphate
Auto-mUb	Auto-monoubiquitylation
B-box2	B-box type 2
Bis-Tris	2-[bis(2-hydroxyethyl)amino]-2-(hydroxymethyl)propane-1,3-diol
BNP	B-type Natriuretic Peptide
CC	Coiled-coil
COS-box	C-terminal subgroup One Signature-box
DNA	Deoxyribonucleic Acid
dNTP	Deoxynucleoside triphosphate
DTT	Dithiothreitol
E1, UBE1	Ubiquitin-activating enzyme
E2, UBE2	Ubiquitin-conjugating enzyme
E3, UBE3	Ubiquitin-protein ligase
EPR	Electron paramagnetic resonance
FnIII	Fibronectin type III
FWD	Forward
HADDOCK	High Ambiguity Driven protein-protein Docking
HCM	Hypertrophic cardiomyopathy
HD	Helical domain
HECT	Homologous to E6AP C-terminus
HEPES	4-(2-Hydroxyethyl)piperazine-1-ethane-sulfonic acid
His-, His-tag	Histidine tag
His-Titin-mUb	His-Titin (A168-A170) monoubiquitylation
HRP	Horseradish peroxidase
Ig	Immunoglobulin-like
IPTG	Isopropyl β-D-1-thiogalactopyranoside
MBP	Maltose-binding protein
MFC	MuRF family-specific motif
MgCl_2_	Magnesium chloride
MID1	Midline-1 protein
MOPS	3-(N-morpholino)propanesulfonic acid
MuRF1	Muscle RING-finger protein-1 protein
NaCl	Sodium chloride
NEB Tm Calculator	New England Biolabs Melting Temperature Calculator
NMD	Nonsense-mediated decay pathway
NuPAGE LDS	NuPAGE Lithium Dodecyl Sulphate
OD	Optical density
PCR	Polymerase chain reaction
PDB	Protein Data Bank
PELDOR	Pulsed electron-electron double resonance
Phyre2	Protein Homology/analogY Recognition Engine V 2.2
PMSF	Phenylmethylsulfonyl fluoride
PRALINE	PRofile ALIgNEment
PVDF	Polyvinylidene fluoride
PyMOL	PyMOL Molecular Graphics System version 2.6.0
RBR	RING-between-RING
REV	Reverse
RING	Really Interesting New Gene
RMSD	Root Mean Square Deviation
RPM	Revolutions per minute
SDS-PAGE	Sodium dodecyl sulphate–polyacrylamide gel electrophoresis
SEM	Standard error of the mean
SMS-Ident and Sim	Sequence Manipulation Suite-Identity and Similarity
TBS-T	1xTris-buffered saline-Tween 20
TCEP	Tris(2-carboxyethyl)phosphine
TRIM18	Tripartite motif-containing 18
TRIM21	Tripartite motif-containing 21
TRIM25	Tripartite motif-containing 25
TRIM63	Tripartite motif-containing 63
Tris-HCl	Tris(hydroxymethyl)aminomethane hydrochloride
UBE2W	Ubiquitin-conjugating Enzyme E2W
UniProt	Universal Protein Resource
UPS	Ubiquitin–proteasome system
WT	Wild type
ZnSO_4_	Zinc sulphate
