# Supplementary material for: HCM-Associated MuRF1 Variants Compromise Ubiquitylation and Are Predicted to Alter Protein Structure
Source: Int J Mol Sci. 2025 Apr 21;26(8):3921. doi: 10.3390/ijms26083921 (PMC12027535; doi:10.3390/ijms26083921)
Supplement: Supplementary file 1 [file ijms-26-03921-s001.zip › ijms-3488491-supplementary.pdf]

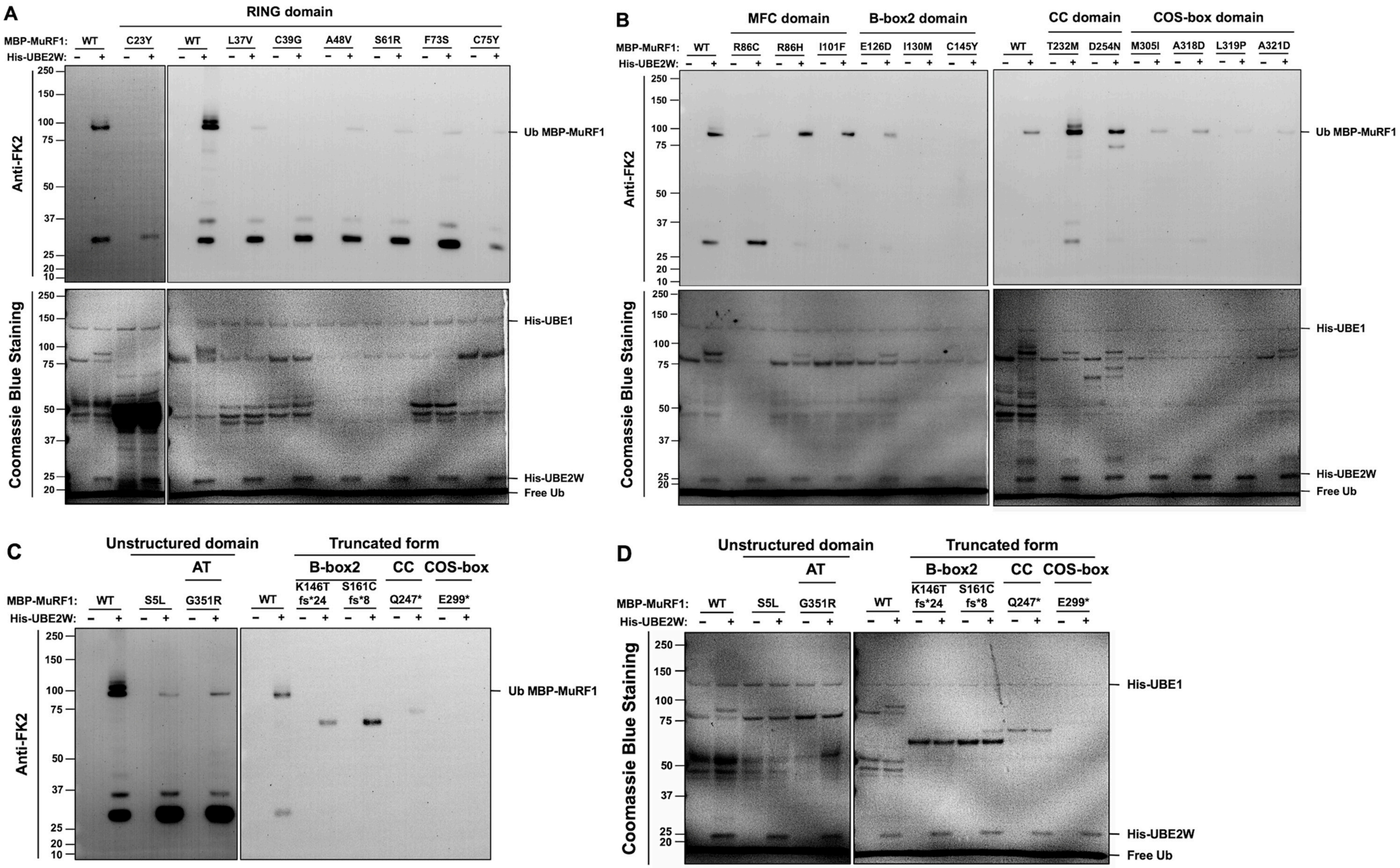

Figure S1. The results of anti-FK2 and Coomassie blue staining of 25 MuRF1 variants in auto-ubiquitylation assay. (A) Anti-FK2 and Coomassie blue staining results of MuRF1 missense variants on RING. (B) Anti-FK2 and Coomassie blue staining results of MuRF1 missense variants on MFC, B-box2, Coiled-coil, and COS-box domains. (C) Anti-FK2 results of unstructured and truncated MuRF1 variants. (D) Coomassie blue staining results of unstructured and truncated MuRF1 variants.

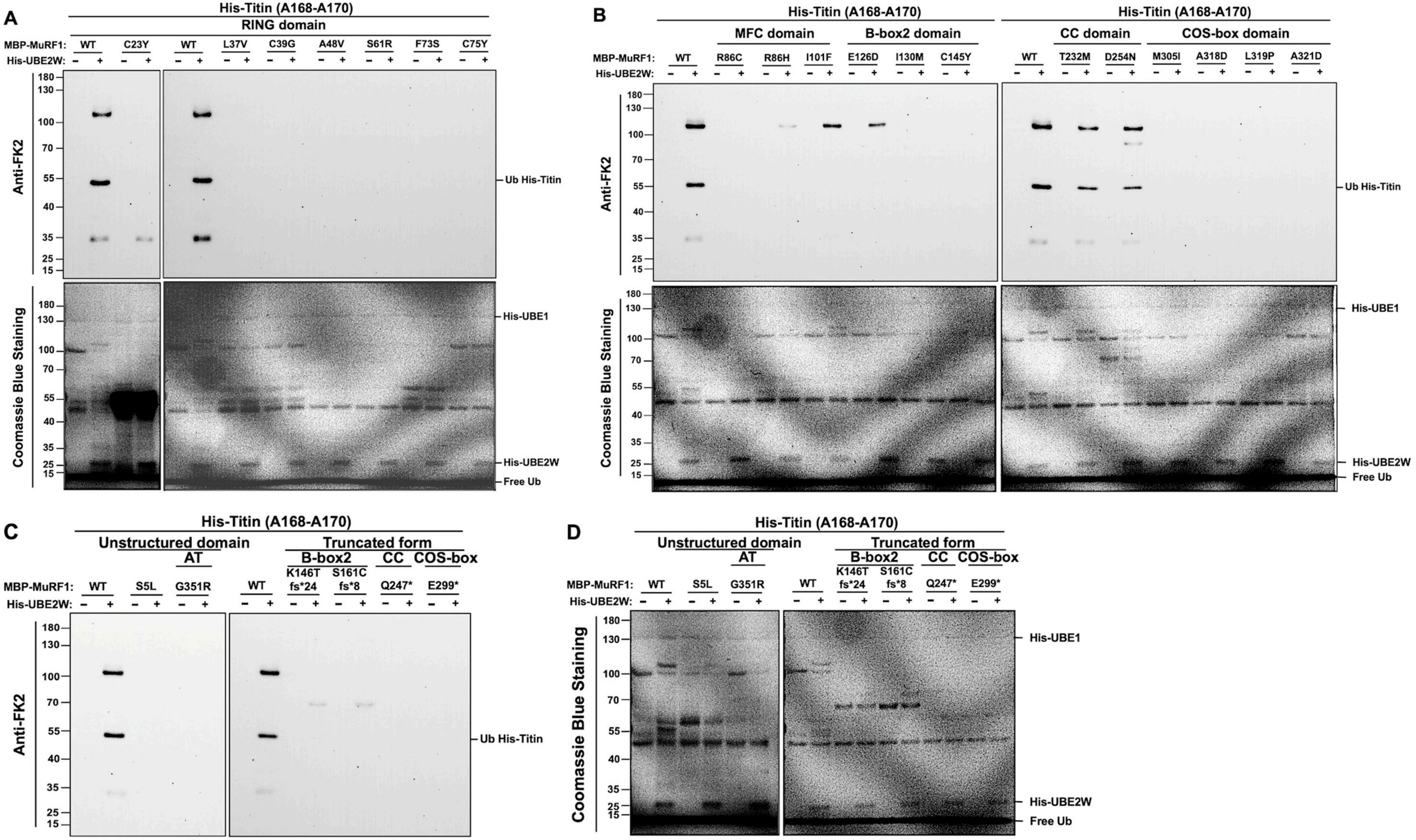

Figure S2. The results of anti-FK2 and Coomassie blue staining of 25 MuRF1 variants in His-Titin (A168-A170) ubiquitylation assay. (A) Anti-FK2 and Coomassie blue staining results of MuRF1 missense variants on RING. (B) Anti-FK2 and Coomassie blue staining results of MuRF1 missense variants on MFC, B-box2, Coiled-coil, and COS-box domains. (C) Anti-FK2 results of unstructured and truncated MuRF1 variants. (D) Coomassie blue staining results of unstructured and truncated MuRF1 variants.

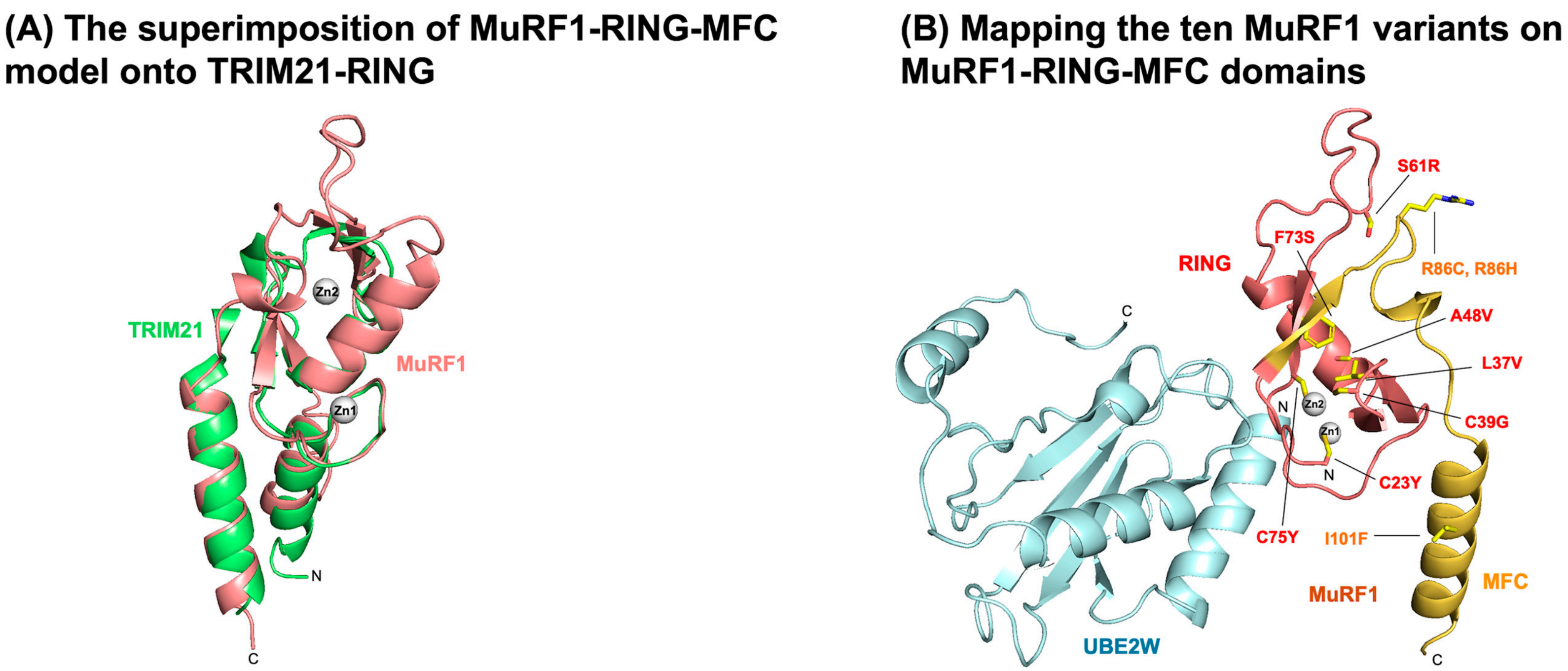

Figure S3. Structural modelling of the MuRF1-RING-MFC domains. (A) The superimposition of the AlphaFold2-derived MuRF1-RING-MFC model (pink) onto the TRIM21-RING structure (green) (PDB ID: 8A58) [26], revealed a good structural alignment with RMSD of 0.729 Å. (B) The HADDOCK-derived model of MuRF1-RING-MFC in complex with UBE2W. Mapping of the ten *MuRF1* variants on the MuRF1 RING and MFC domains, indicates that none of these variants are located in the direct putative UBE2W binding interface.

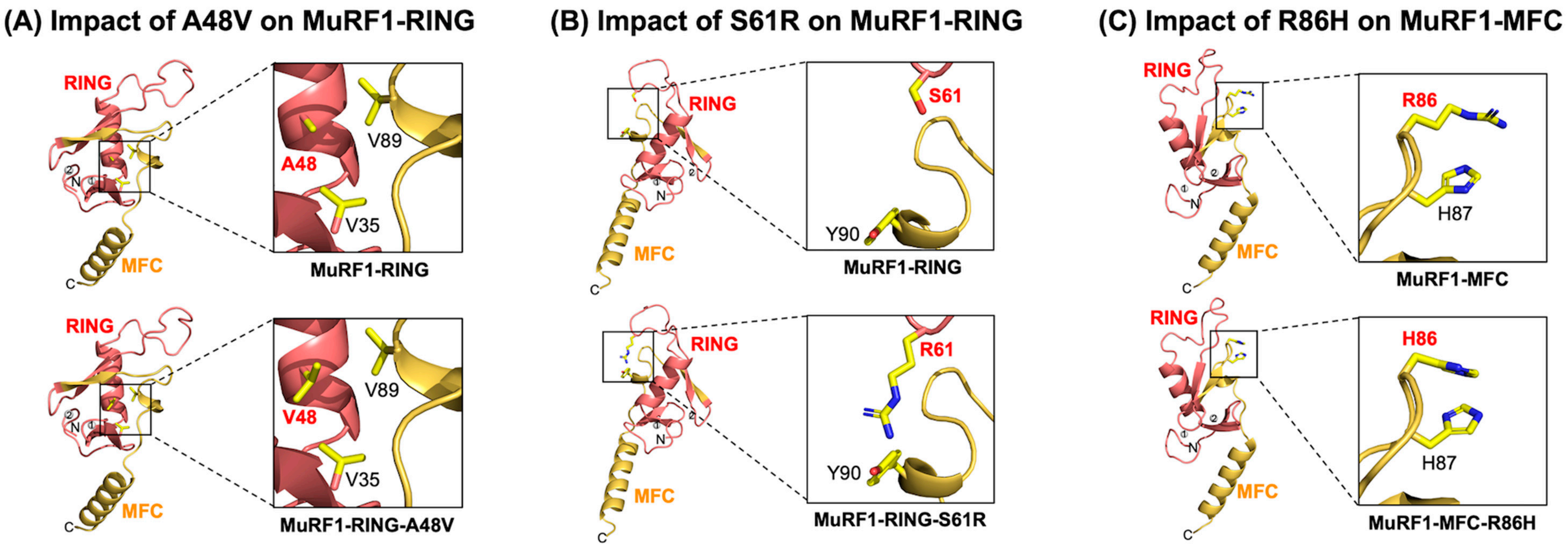

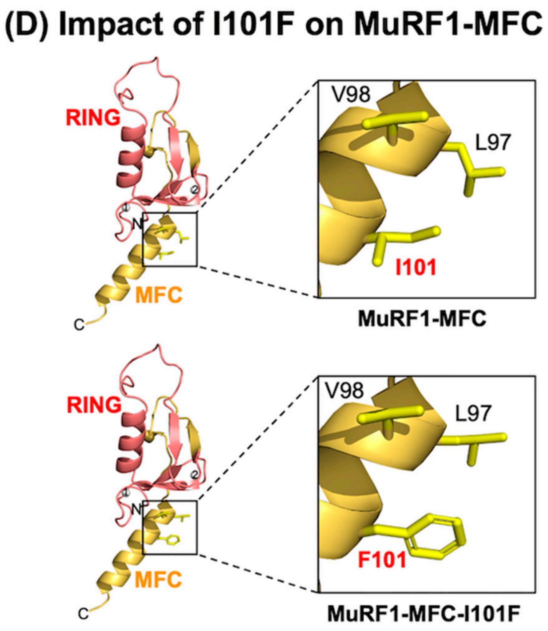

Figure S4. Assessing the impact of the A48V, S61R, R86H, and I101F variants on the MuRF1 RING and MFC domain structures. (A) Ribbon representation of the AlphaFold2-derived molecular model of the MuRF1-RING-MFC structure, showing A48 mediates non-polar contacts with V35 and V89 (top panel). The introduction of the A48V variant is predicted to increase the number of non-polar interactions with neighbouring residues (lower panel). (B) Ribbon representation of the AlphaFold2-derived molecular model of the MuRF1-RING-MFC structure, highlighting S61, does not contribute to the structure stability of the RING domain (top panel). The introduction of the S61R variant is predicted to result in cation-π interaction between R61 and Y90 (lower panel). (C) Ribbon representation of the AlphaFold2-derived molecular model of the MuRF1-RING-MFC structure, showing R86 mediates cation-π interaction with H87 to stabilise the MFC domain (top panel). The introduction of the R86H variant is predicted to result in a π-π stacking interaction with H87 (lower panel). (D) Ribbon representation of the AlphaFold2-derived molecular model of the MuRF1-RING-MFC structure, highlighting I101 mediates minimal interactions with nearby residues (top panel). The introduction of the I101F variant is likely to form similar contacts suggesting that it exerts no effect on the MuRF1-MFC structure (lower panel). Close-up views of relevant interactions are provided in boxes.

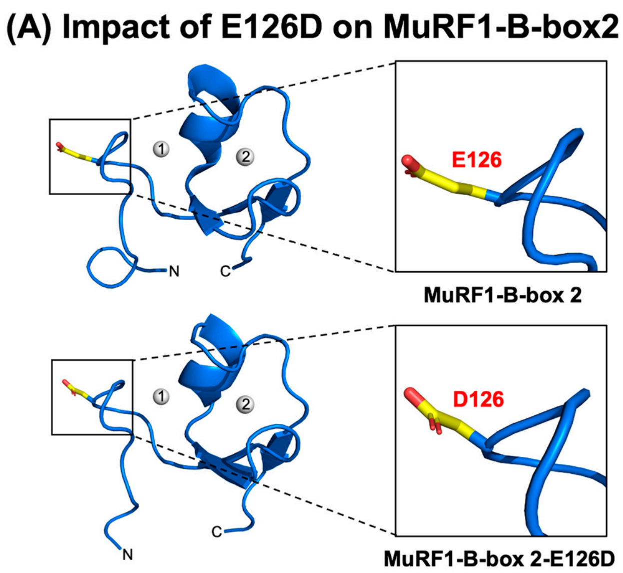

Figure S5. Examining the effect of the E126D variant on the MuRF1-B-box2 domain structure. (A) Ribbon representation of the AlphaFold2-derived molecular model of the MuRF1-B-box2 domain, showcasing that the solvent-exposed E126 does not contribute to the structure stability of the B-box2 domain (top panel). Introduction of the E126D variant is predicted to be structurally neutral (below panel). Close-up views of relevant interactions are provided in boxes.

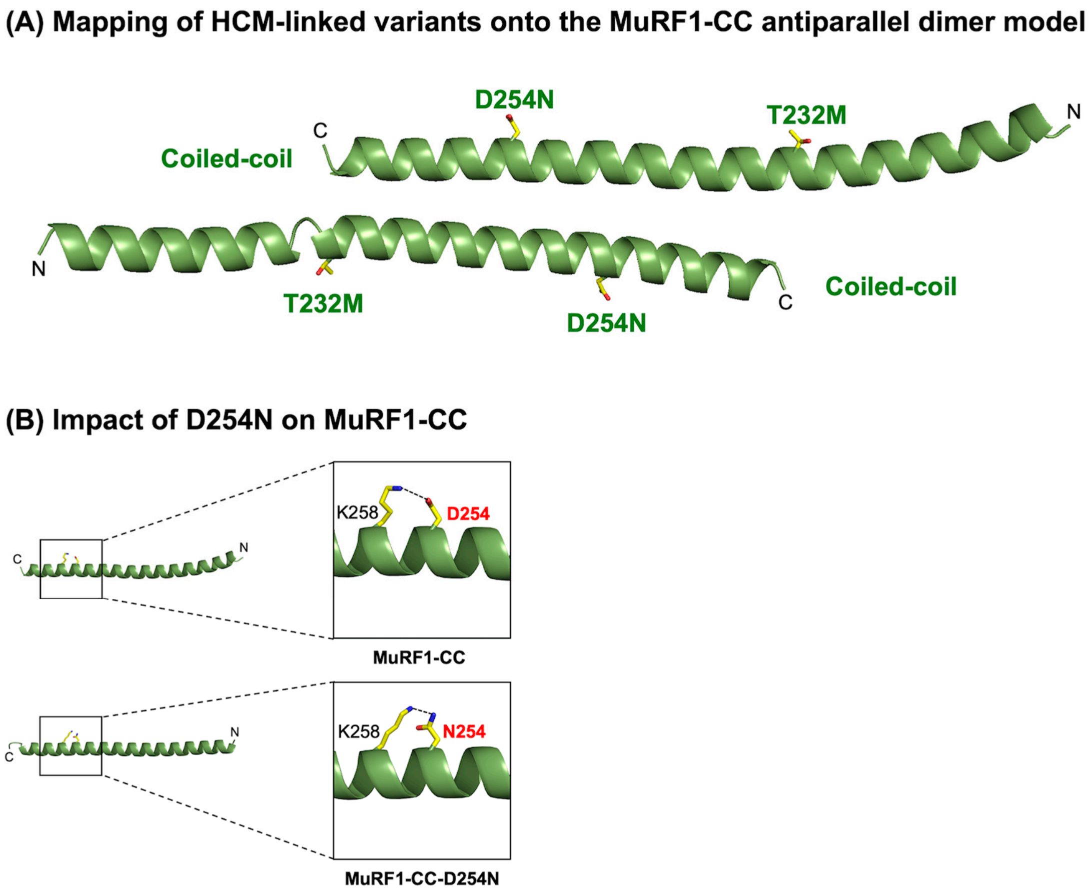

Figure S6. Assessing the impact of the D254N mutation on the MuRF1-CC domain structure. (A) Mapping of HCM-linked variants onto the MuRF1-CC antiparallel dimer model, reveals that both variants do not contribute to stabilising the dimer interface. (B) Ribbon representation of the AlphaFold2-derived molecular model of the MuRF1-CC domain, illustrating that D254 mediates a salt bridge with K258 to stabilise the CC domain (top panel). The N254 variant is predicted to form a compensatory hydrogen bond with K258, which may stabilise the CC domain (below panel). Close-up views of relevant interactions are provided in boxes. The black dashed lines represent polar interactions.

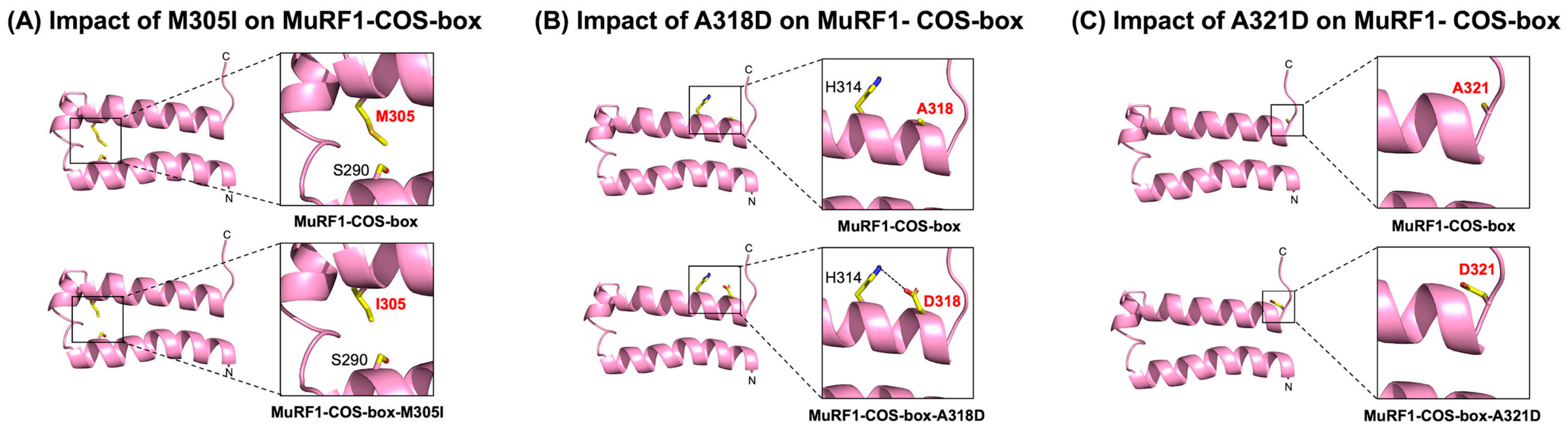

Figure S7. Examining the impact of the M305I, A318D and A321D variants on the MuRF1-COS-box domain structure. (A) Ribbon representation of the Phyre2-derived molecular model of the MuRF1-COS-box domain, showing that M305 mediates multiple contacts with S290 and T309 to stabilise COS-box domain core (top panel). Introduction of the M305I is anticipated to maintain interactions in this region (lower panel). (B) Ribbon representation of the Phyre2-derived molecular model of the MuRF1-COS-box domain, highlighting that A318 mediates minimal contributions to stabilising the COS-box domain (top panel). Introduction of the A318D variant is predicted to strengthen interactions by forming a hydrogen bond with H314 (lower panel). (C) Ribbon representation of the Phyre2-derived molecular model of the MuRF1-COS-box domain, illustrating that A321 does not contribute to the structural stability of the COS-box domain (top panel). Introduction of the A321D variant is predicted to exert structurally neutral effects (below panel). Close-up views of relevant interactions are provided in boxes. The black dashed line represents a hydrogen bond.

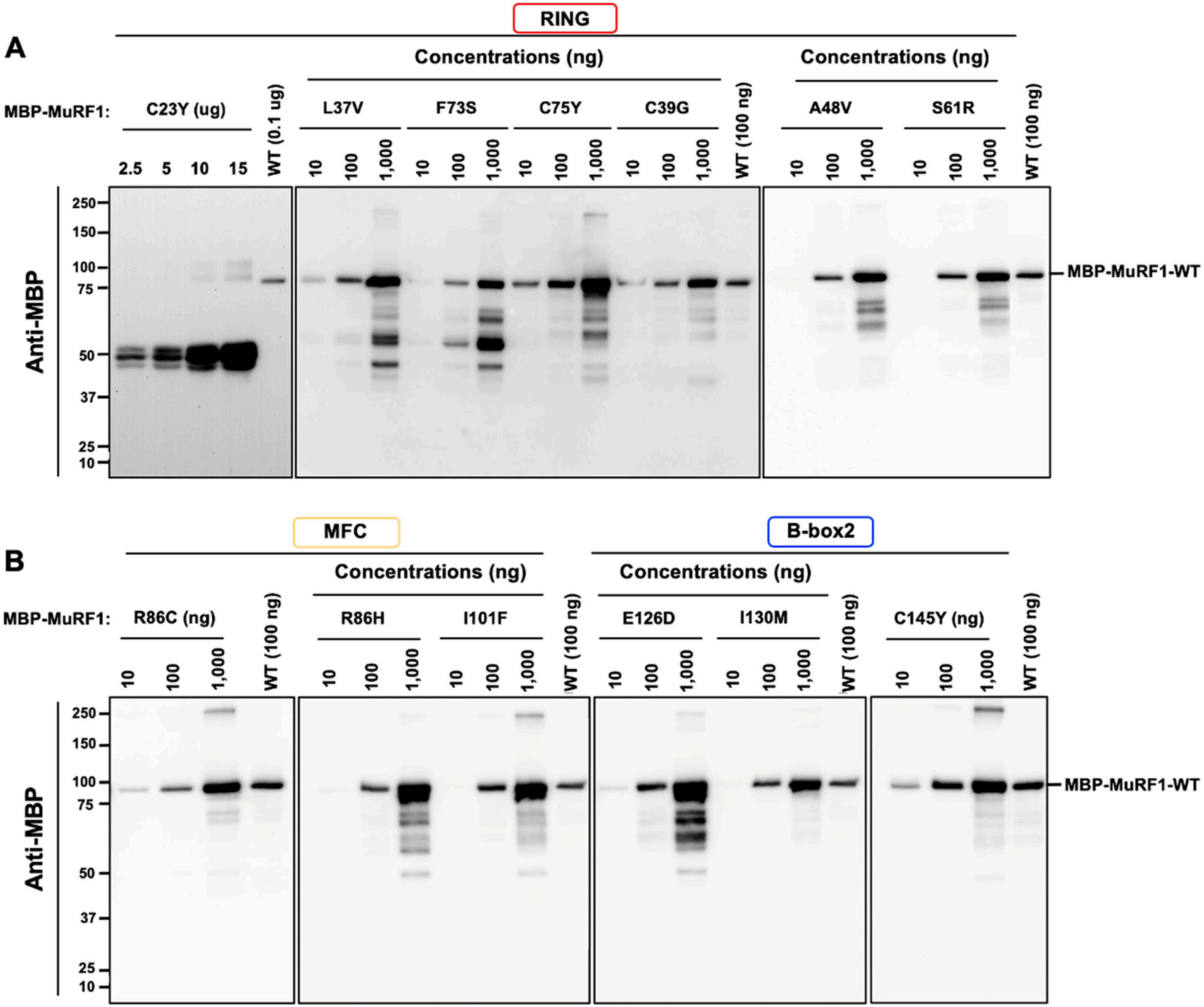
A
RING
MBP-MuRF1:
C23Y (ug)
2.5
5
10
15
WT (0.1 ug)
Concentrations (ng)
L37V
F73S
C75Y
C39G
10
100
1,000
WT (100 ng)
A48V
S61R
Anti-MBP
250
150
100
75
50
37
25
10
MBP-MuRF1-WT
B
MFC
B-box2
R86C (ng)
R86H
I101F
E126D
I130M
C145Y (ng)

C

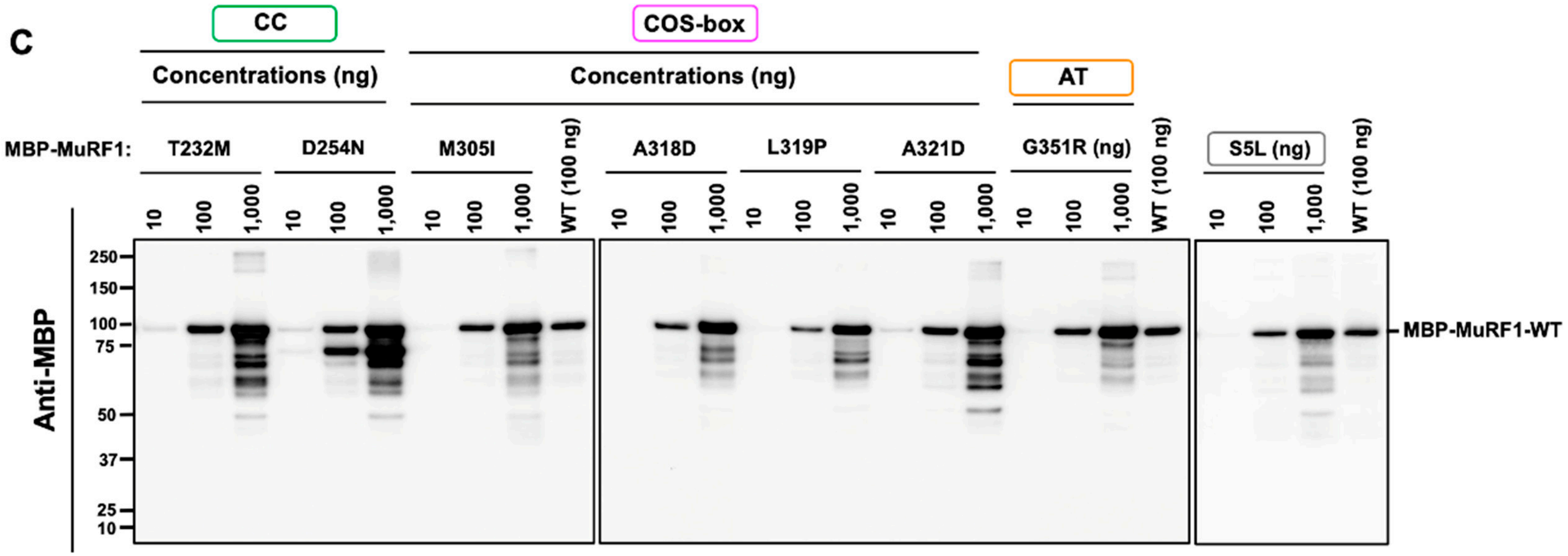
CC
COS-box
Concentrations (ng)
Concentrations (ng)
AT
MBP-MuRF1:
T232M
D254N
M305I
A318D
L319P
A321D
G351R (ng)
S5L (ng)
WT (100 ng)
10
100
1,000
Anti-MBP
250
150
100
75
50
37
25
10
MBP-MuRF1-WT

D

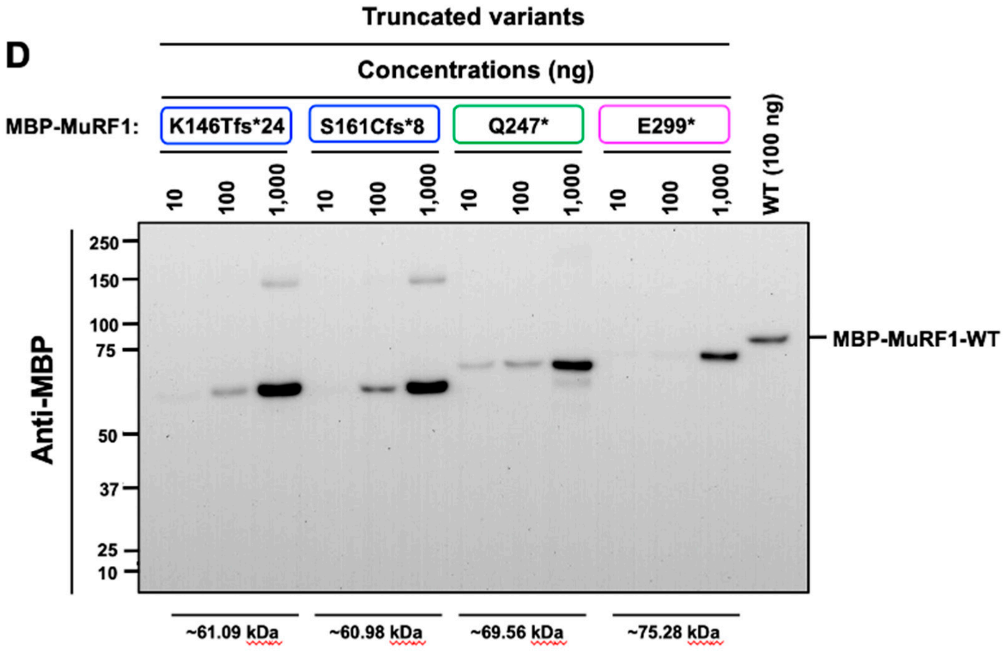
Truncated variants
Concentrations (ng)
MBP-MuRF1:
K146Tfs*24
S161Cfs*8
Q247*
E299*
WT (100 ng)
10
100
1,000
Anti-MBP
250
150
100
75
50
37
25
10
MBP-MuRF1-WT
~61.09 kDa
~60.98 kDa
~69.56 kDa
~75.28 kDa

Figure S8. The stability and optimal loading of 25 recombinant MuRF1 proteins were evaluated. Among these, 20 missense variants demonstrated optimal loading at a concentration of 100 ng. However, the missense variant C23Y exhibited a significant degradation product at approximately 50 kDa. The truncated variants (K146Tfs*24, S161Cfs*8, Q247*, E299*) displayed optimal loading at a concentration of 1,000 ng compared to the wild type (WT). The results are presented as follows: A) RING domain; B) MFC and B-box2 domains; C) Coiled-coil, COS-box, and unstructured domains; D) The four truncated variants located in different domains show truncated forms.

Table S1. The clinical relevance of the 25 *TRIM63* variants was summarised from seven published papers. The effects of these 25 variants on the disease were evaluated by analysing their occurrence likelihood using MAF values from gnomAD v2.1.1, along with their impact on protein function assessed through the PolyPhen-2 and SIFT platforms.

No.	Protein (Disease)	Domain	Type of Mutation	Additional genetic variants in patient (s)	Reference	Pathogenicity prediction of 25 *TRIM63* variants			Amino acid conservation Across	
						MAF on GnomAD	PolyPhen-2 (score)	SIFT (score)	MuRF1 species	MuRF family
1	S5L (HCM)	N terminal	Missense	None	[9]	$2.12x10^{-5}$	PRO (0.985)	T (0.38)	Yes	No
2	C23Y (HCM)	RING	Missense	(RYR2-H464Q/ TTN-G16395E)	[23]	$3.18x10^{-5}$	PRO (1.000)	D (0.00)	Yes	Yes
3	L37V (HCM)	RING	Missense	MuRF1-C75Y	[23]	Absent	PRO (1.000)	D (0.04)	Yes	Yes
4	C39G (HCM)	RING	Missense	MuRF1-S161Cfs*8	[24]	Absent	PRO (1.000)	D (0.00)	Yes	Yes
5	A48V (HCM)	RING	Missense	None	[19]	$1.32x10^{-3}$	PRO (0.998)	T (0.23)	Yes	Yes
6	S61R (HCM)	RING	Missense	MYH7-K1757E	[9]	Absent	BEN (0.079)	T (0.05)	No	No
7	F73S (HCM)	RING	Missense	None	[9]	$4.63x10^{-5}$	PRO (1.000)	D (0.00)	Yes	Yes
8	C75Y (HCM)	RING	Missense	MuRF1-L37V/ MuRF1-Q247*/ TCAP-A118V	[23, 24]	$9.95x10^{-5}$	PRO (1.000)	D (0.00)	Yes	Yes
9	R86C (HCM)	MFC	Missense	None	[9]	$2.00x10^{-5}$	PRO (0.980)	D (0.00)	Yes	Yes
10	R86H (HCM)	MFC	Missense	MYBPC3-E258K	[9]	$1.19x10^{-5}$	PRO (1.000)	D (0.03)	Yes	Yes

No.	Protein (Disease)	Domain	Type of Mutation	Additional genetic variants in patient (s)	Reference	Pathogenicity prediction of 25 *TRIM63* variants			Amino acid conservation Across	
						MAF on GnomAD	PolyPhen-2 (score)	SIFT (score)	MuRF1 species	MuRF family
11	I101F (HCM)	MFC	Missense	None	[9]	Absent	PRO (0.987)	D (0.01)	Yes	Yes
12	E126D (HCM)	B-box 2	Missense	None	[9]	$7.56x10^{-5}$	BEN (0.216)	D (0.04)	Yes	Yes
13	I130M (HCM)	B-box 2	Missense	None	[19]	$4.77x10^{-5}$	POS (0.880)	D (0.01)	Yes	Yes
14	C145Y (RCM)	B-box 2	Missense	DSC2-V129I	[23]	$2.39x10^{-5}$	PRO (1.000)	D (0.00)	Yes	Yes
15	K146Tfs*24 (HCM)	B-box 2	Frameshift	MYBPC3-Y213*/ MuRF1-Q247*/ DTNA-V174I	[23]	Absent	-	-	Yes	Yes
16	S161Cfs*8 (HCM)	B-box 2	Frameshift	MuRF1-C39G/ TTN-Y10050C/ /TPM1 c.375-3C>T	[23, 24]	$4.25x10^{-5}$	-	-	Yes	No
17	T232M (HCM)	CC	Missense	MYBPC3, c.3491-1G>A	[9]	$1.59x10^{-5}$	PRO (0.990)	T (0.08)	Yes	No
18	Q247* (HCM)	CC	Nonsense	MuRF1-C75Y/ MuRF1-K146Tfs*24/ OBSCN-L7883R/ KCNE1-R36H/ TCAP-C38F/ DTNA-V174I/ MuRF3-D106N	[19-24]	$6.79x10^{-4}$	-	-	No	No
19	D254N (HCM)	CC	Missense	MYH7-K1242fs	[9]	Absent	BEN (0.161)	D (0.01)	No	No

No.	Protein (Disease)	Domain	Type of Mutation	Additional genetic variants in patient (s)	Reference	Pathogenicity prediction of 25 *TRIM63* variants			Amino acid conservation Across	
						MAF on GnomAD	PolyPhen-2 (score)	SIFT (score)	MuRF1 species	MuRF family
20	E299* (HCM)	COS-box	Nonsense	MYL3-E49D	[9]	Absent	-	-	Yes	Yes
21	M305I (HCM)	COS-box	Missense	None	[9]	Absent	POS (0.898)	T (0.12)	Yes	Yes
22	A318D (HCM)	COS-box	Missense	MYH7-R453C	[9]	3.98×10^{-6}	POS (0.602)	D (0.01)	Yes	No
23	L319P (HCM)	COS-box	Missense	COQ2-Q94Hfs*78	[23]	9.54×10^{-5}	PRO (1.000)	D (0.00)	Yes	No
24	A321D (HCM)	COS-box	Missense	MYBPC3-R160W	[9]	Absent	BEN (0.000)	T (0.32)	Yes	No
25	G351R (HCM)	AT	Missense	MYL2-R58Q	[9]	8.62×10^{-6}	BEN (0.001)	T (0.48)	Yes	No

The MAF value is considered to be pathogenic when the value is lower than 10^{-4}. The PolyPhen-2 score close to 1 is predicted to be damaging, which means impairing protein function; PRO, probably damaging; POS, possibly damaging; BEN, benign damaging. The SIFT score of lower than 0.05 is predicted to be deleterious, which means harming protein function; D, deleterious; T, tolerated. The amino acid conservation between MuRF1 species (Human, Rat, Mouse) and MuRF family (MuRF1, MuRF2, and MuRF3) in each variant is also added in this table. The amino acid conservation of MuRF1 in humans is classified as "Yes" if the amino acid is conserved in both rat and mouse, or in both MuRF2 and MuRF3. Conversely, it is classified as "No" if the amino acid is not conserved in either rat or mouse, nor in either MuRF2 or MuRF3.

Table S2. Nucleotide changes and location of the 25 HCM-associated *TRIM63* variants

No.	Nucleotide change	Amino acid change	Domain
1	c.14C>T	S5L	N-terminal
2	c.68G>A	C23Y	RING
3	c.109T>G	L37V	RING
4	c.115T>G	C39G	RING
5	c.143C>T	A48V	RING
6	c.183C>A	S61R	RING
7	c.218T>C	F73S	RING
8	c.224G>A	C75Y	RING
9	c.256C>T	R86C	MFC
10	c.257G>A	R86H	MFC
11	c.301A>T	I101F	MFC
12	c.378A>T	E126D	B-box2
13	c.390C>G	I130M	B-box2
14	c.434G>A	C145Y	B-box2
15	c.437_442delAGGTGTinsCC	K146Tfs*24	B-box2
16	c.481_482delAG	S161Cfs*8	B-box2
17	c.695C>T	T232M	CC
18	c.739 C>T	Q247*	CC
19	c.760G>A	D254N	CC
20	c.895G>T	E299*	COS-box
21	c.915G>T	M305I	COS-box
22	c.953C>A	A318D	COS-box
23	c.956T>C	L319P	COS-box
24	c.962C>A	A321D	COS-box
25	c.1051G>A	G351R	AT

Table S3. Primer sequences are used in single-point and double mutagenesis of *TRIM63*.

No.	Primer name of *TRIM63*	Primer sequences (5′ to 3′)
1	S5L_FWD	TTATAAGTtGAGCCTGATCCAGGATGGGAATCCCAT
	S5L_REV	TCAGGCTCaACTTATAATCCATaCCGCCCTGAAAATACA
2	C23Y_FWD	AGCTGATCTaCCCTATCTGCCTGGAGATGTTTACCAAGCCAG
	C23Y_REV	GATAGGGtAGATCAGCTGCTTCTCCAAGTTCTCCATGGGATTCC
3	L37V_FWD	GTCATCgTGCCGTGCCAGCACAACCTGTGCC
	L37V_REV	CACGGCAcGATGACCACTGGCTTGGTAAACATCTCC
4	C39G_FWD	TGCCGgGCCAGCACAACCTGTGCCGGAAG
	C39G_REV	CTGGCcCGGCAaGATGACCACTGGCTTGGTAAAC
5	A48V_FWD	GAAGTGTGtCAATGACATCTTCCAGGCTGCAAATCCCTAC
	A48V_REV	TGTCATTGaCACACTTCCGGCACAGGTTGTGCTGG
6	S61R_FWD	ACCAGaCGGGGCAGCTCAGTGTCCATGTCTGGAG
	S61R_REV	CCCCGtCTGGTCCAGTAGGGATTTGCAGCCTGG
7	F73S_FWD	CCGTTcCCGCTGCCCCACCTGCCGCCACGAGGTGATCATGGATCGTCA
	F73S_REV	AGCGGgAACGGCCTCCAGACATGGACACTGAGCTG
8	C75Y_FWD	TCCGCTaCCCCACCTGCCGCCACGAGGTGATCATGGATCGTCA
	C75Y_REV	TGGGGtAGCGGAAACGGCCTCCAGACATGG
9	R86C_FWD	GGATtgTCACGGAGTGTACGGCCTGCAGAG
	R86C_REV	TCCGTGAcaATCCATGATCACCTCGTGGCGG
10	R86H_FWD	GGATcaTCACGGAGTGTACGGCCTGCAGAG
	R86H_REV	TCCGTGAtgATCCATGATCACCTCGTGGCGG
11	I101F_FWD	GGAGAACtTCATCGACATCTACAAACAGGAGTGCTCCAG
	I101F_REV	GTCGATGAaGTTCTCCACCAGCAGGTTCCTCTGC
12	E126D_FWD	GGAGCACGAtGATGAGAAAATCAACATCTACTGTCTCACGTGTGAGG
	E126D_REV	CTCATCaTCGTGCTCCTTGCACATGGGGTGACTGC
13	I130M_FWD	GAGAAAATgAACATCTACTGTCTCACGTGTGAGGTGCCC
	I130M_REV	GACAGTAGATGTTcATTTTCTCATCtTCGTGCTCCTTGCACATG
14	C145Y_FWD	CCATGTaCAAGGTGTTTGGGATCCACAAGGCCTG
	C145Y_REV	CACCTTGtACATGGAGCAGGTGGGCACCTCA
15	K146Tfs*24_FWD	CATGTGCACCTTGGGATCCACAAGGCCTGCGAGG
	K146Tfs*24_REV	CCCAAGGTGCACATGGAGCAGGTGGGCACCTCA
16	S161Cfs*8_FWD	CAGTGTCTTCCAGGGACAAAAGACTGAACTGAATAACTGTATCTCCATGCTGG

No.	Primer name of *TRIM63*	Primer sequences (5′ to 3′)
	S161Cfs*8_REV	TGTCCCTGGAAGACACTGCAATGGGGCCACCTCGCA
17	T232M_FWD	CGGATCAtGCAGGAGCAGGAGAAAAAGCTTAGCTTCATCGAGG
	T232M_REV	TCCTGCaTGATCCGCTGCAGCAACTCACTTTTCTTCTCATCC
18	Q247*_FWD	CCTCATCtAGCAGTACCAGGAGCAGCTGgACAAGTC
	Q247*_REV	GTACTGCTaGATGAGGGCCTCGATGAAGCTAAGCTTTTTCTC
19	D254N_FWD	CAGCTGaACAAGTCCACAAAGCTGGTGGAAACTGCC
	D254N_REV	GGACTTGTtCAGCTGCTCCTGGTACTGCTGGATGAGG
20	E299*_FWD	GGGGAAGACAtAGCAGGGCTTTGAGAACATgGACTTCTTTACTTTGG
	E299*_REV	TGCTaTGTCTTCCCCAGCTGGCAGCCCTTGGAA
21	M305I_FWD	TGAGAACATtGACTTCTTTACTTTGGATTTAGAGCACATAGCAGACG
	M305I_REV	AGAAGTCaATGTTCTCAAAGCCCTGCTcTGTCTTCC
22	A318D_FWD	AGACGaCCtGAGAGcCATTGACTTTGGGACAGATGAG
	A318D_REV	TCTCaGGtCGTCTGCTATGTGCTCTAAATCCAAAGTAAAGAAGTCc
23	L319P_FWD	AGACGcCCcGAGAGcCATTGACTTTGGGACAGATGAG
	L319P_REV	TCTCgGGgCGTCTGCTATGTGCTCTAAATCCAAAGTAAAGAAGTCc
24	A321D_FWD	CCtGAGAGaCATTGACTTTGGGACAGATGAGGAAGAGGAAGAATTC
	A321D_REV	GTCAATGtCTCTCaGGgCGTCTGCTATGTGCTCTAAATCCAAAG
25	G351R_FWD	GAAGAAaGACACCAGTAAgcGGCCGCTCAATT
	G351R_REV	CTGGTGTCtTTCTTCCTTCCCTTCTGTGGACTCTTC

Table S4. Bioinformatic tools used in this study

NO.	Tool	Detail	Source	Date accessed
1	Benchling	-	https://www.benchling.com	01/03/22 - 30/04/23
2	NEB Tm Calculator	New England Biolabs Melting Temperature Calculator	https://tmcalculator.neb.com/#!/main	01/01/22 - 31/01/22
3	SIFT	Sorting Intolerant from Tolerant	https://sift.bii.a-star.edu.sg	06/11/23
4	PolyPhen-2	Polymorphism Phenotyping v2	http://genetics.bwh.harvard.edu/pph2/	22/11/23
5	MAF	the minor allele frequency (MAF) value in gnomAD v2.1.1	https://gnomad.broadinstitute.org	14/11/24
6	IBS 2.0	Illustrator for Biological Sequences, version 2.0	https://ibs.renlab.org/#/home	01/06/24 - 31/07/24
7	PDB	Protein Data Bank	https://www.rcsb.org	10/06/24 - 31/12/24
8	UniProt	Universal Protein Resource	https://www.uniprot.org	10/06/24 - 31/12/24
9	PRALINE	PRofile ALIgNEment	https://www.ibi.vu.nl/programs/pralinewww/	01/07/24 - 31/07/24
10	SMS-Ident and Sim	Sequence Manipulation Suite-Identity and Similarity	https://www.bioinformatics.org/sms2/ident_sim.html	01/07/24 - 31/07/24
11	ColabFold	AI tool based on AlphaFold2 algorithm	https://colab.research.google.com/github/sokrypton/ColabFold/blob/main/AlphaFold2.ipynb	01/06/24 - 31/10/24
12	Phyre2	**P**rotein Homology/analog**Y** **R**ecognition **E**ngine V 2.2	http://www.sbg.bio.ic.ac.uk/~phyre2/html/page.cgi?id=index	01/06/24 - 31/10/24
13	HADDOCK	High Ambiguity Driven protein-protein Docking 2.4	https://rascar.science.uu.nl/haddock2.4/	01/07/24 - 31/07/24
14	PyMOL	PyMOL Molecular Graphics System Version 2.6.0 Copyright (C) Schrödinger, LLC	https://pymol.org	10/06/24 - 31/12/24
